# An inferential framework for biological network hypothesis tests

**DOI:** 10.1186/1471-2105-14-94

**Published:** 2013-03-14

**Authors:** Phillip D Yates, Nitai D Mukhopadhyay

**Affiliations:** 1 Pfizer Global Research and Development, Groton, CT, USA; 2 Department of Biostatistics, Virginia Commonwealth University, Richmond, VA, USA

## Abstract

**Background:**

Networks are ubiquitous in modern cell biology and physiology. A large literature exists for inferring/proposing biological pathways/networks using statistical or machine learning algorithms. Despite these advances a formal testing procedure for analyzing network-level observations is in need of further development. Comparing the behaviour of a pharmacologically altered pathway to its canonical form is an example of a salient one-sample comparison. Locating which pathways differentiate disease from no-disease phenotype may be recast as a two-sample network inference problem.

**Results:**

We outline an inferential method for performing one- and two-sample hypothesis tests where the sampling unit is a network and the hypotheses are stated via network model(s). We propose a dissimilarity measure that incorporates nearby neighbour information to contrast one or more networks in a statistical test. We demonstrate and explore the utility of our approach with both simulated and microarray data; random graphs and weighted (partial) correlation networks are used to form network models. Using both a well-known diabetes dataset and an ovarian cancer dataset, the methods outlined here could better elucidate co-regulation changes for one or more pathways between two clinically relevant phenotypes.

**Conclusions:**

Formal hypothesis tests for gene- or protein-based networks are a logical progression from existing gene-based and gene-set tests for differential expression. Commensurate with the growing appreciation and development of systems biology, the dissimilarity-based testing methods presented here may allow us to improve our understanding of pathways and other complex regulatory systems. The benefit of our method was illustrated under select scenarios.

## Background

Networks, a construct emphasizing the interrelations between objects, have application in studying human behaviour, mathematics, physics, econometrics, etc. With the introduction of microarrays and other high-throughput systems, networks increasingly provide a means to organize and study the interdependencies of genes, proteins, metabolites, etc. Gene transcription/regulatory networks, metabolic pathways, protein-protein interaction systems (PPIs), signal transduction pathways, and phylogenetic trees are established tools in biology. Prone-to-noise experiments are routinely coupled with computational algorithms to infer relationships. Given the empirical reliance on uncertain data it seems natural to ask, “Do these networks differ from one another?” This paper outlines and demonstrates an inferential process for performing one- and two-sample hypothesis tests when the sample data are biological networks.

Numerous books are devoted to networks. Bornholdt *et al.*[1] and Junker *et al*. [2] provide a broad introduction to networks with application to biology, e.g., correlation profiles and motifs, network behaviour in nematode development, etc. Emmert-Streib *et al*. [3] is a collection devoted to inferring microarray-based networks. Kolaczyk [4] appears to be the first statistics text devoted solely to networks. Brandes *et al*. [5] provide an overview of analysis methods from a computer science perspective.

Testing graphs is not trivial; comparing two static graphs is conceptually different from comparing a sample of stochastic graphs under one or more treatments. Defining a suitable null probability model for a network is a difficult consideration [6]. Erdős-Rényi random graphs, where the probability of an edge between any two vertices is a fixed constant *p*, play an important conceptual role in our understanding of graphs [7]. The use of these graphs in forming statistical tests has received some criticism [6]. Chung *et al*. [8] explore a complex hybrid-graph model to mimic observed small-world networks. Simple models, e.g., random, scale-free, or small-world graphs, etc., although useful for comparing features across a class of networks, may have less utility for weighted biological networks. Schwöbbermeyer [9], in discussing network motifs, makes a troubling comment regarding the formation of actual biological networks, “A single network generation mechanism may not be sufficient to resemble the structure of these networks.” This difficulty in defining a suitably rich or ‘realistic’ parametric model poses a challenge in forming a hypothesis testing procedure for network parameters. Exponential random graph models (ERGMs), referred to as p* models in social network analysis, offer a theoretical model for stochastic networks and have been used in biological applications [10]. ERGM parameterizations can contain attractive motif-like structures. But, these models do not typically assume that the nodes of a graph are aligned and suffer from model degeneracy concerns [4]. Motif frequencies have been used to compare biological networks via a statistical test [9]. Wiuf *et al*. [11] outline a full-likelihood probability model approach to estimating the parameters of a *C. elegans* protein interaction growth model. Cardelli [12] suggests that qualitative models provide more insight than quantitative models due to parameter estimation and criticality concerns. In contrast, Steinhauser *et al*. [13] argue and demonstrate that correlation networks, a form of weighted network, provide more understanding of cellular systems relative to qualitative edge/no-edge models or node-based cell inventory quantitative models. Unlike traditional parameter-centric testing procedures, this dichotomy suggests that a testing procedure might benefit from distinguishing structural (edge) distinctions from weight (quantitative) distinctions. The methods presented here will accommodate this need for a flexible model.

Approaches for both two- and one-sample comparisons occur in the literature. Faust *et al*. [14] use a combination of p* models and correspondence analysis to compare networks. Banks *et al*. [15], using a symmetric difference based on a Hamming distance, outline an estimation/hypothesis testing approach for labeled unweighted loop-free graphs. Sanil *et al*. [16] extend [15] to networks whose edge set evolves in time. Kahlem *et al*. [17], in a reverse engineering application, suggest three approaches (experimentally testable perturbations, training/validation test sets, using synthetic system data) for comparing two networks that conceptually differs from the approach pursued here. Stolovitzky *et al*. [18] propose ROC and precision-recall curves as the method-of-choice for validating inferred models, a motivating example for a one-sample comparison. Chen *et al*. [19] use an additive element-wise score to compare a gene regulatory network estimate to a known network, a parallel to the one-sample procedure developed here.

While our focus here is on forming statistical comparisons of network model parameters, computer scientists can divide the graph comparison problem into two areas – exact graph matching and graph similarity [5]. Graph similarity, tailored to deal with errors in the network data, has been addressed with three broad strategies: compare the difference of path lengths using all pairs of vertices, locate the maximal common subgraph between the two graphs, or use an edit distance. Edit distance, which motivated and resembles the dissimilarity index proposed here, has been used in string matching applications and uses graph operations, e.g., node/edge insertions/deletions, to transform one graph into a second graph [5]. Insertions/deletions can be implemented using set operators. Xulvi-Brunet *et al*. [20] propose a bootstrapped degree of similarity via union/intersection operations. Gill *et al*. [21] combine a partial least squares-based connectivity score with an intersection/union measure to test for differential modular structures via permutation. Expanding beyond strings to motif- or neighbourhood-like objects has demonstrated biological benefit. Li *et al*. [22] extend a node-based topological overlap dissimilarity to assist in defining relevant gene neighbourhoods. Chen *et al*. [19] capture a study where the statistical accuracy of protein function prediction was improved by incorporating information beyond the protein’s immediate neighbours in the network. Such precedent helped motivate the definition of our dissimilarity measure. Graph matching is typically limited to unlabeled graphs. We assume here that the graphs are labeled, an assumption critical for computational and interpretation reasons.

The use and analysis of weighted (correlation-based) networks is increasingly relevant in biological applications. Zhang *et al*. [23], in a close parallel to pure correlation-based networks, form weighted gene co-expression networks. Langfelder *et al*. [24], as part of an R package for analyzing weighted correlation networks, provide several measures for comparing network topologies. Here, we utilize network models based on two forms of correlation matrices. Anderson [25] contains large-sample statistical tests for (partial) correlation coefficients, canonical correlations, and various tests for covariance matrices. Anderson [26] uses a distance-based dissimilarity measure combined with a permutation procedure to compare dispersion matrices.

Resampling methods are common in network analysis, e.g., see [27], and will be used extensively here. Their use in validating network models is less common. Perkins [28] uses cross-validation and resampling methods for validating a gap gene development model. Toh *et al*. [29] use bootstrap samples to assess edge reliability in a partial correlation network. Emmert-Streib [30] combines a permutation-based procedure with a graph-edit distance to compare disease pathways. Xiong *et al.*[31] use a permutation procedure to test the largest element-wise difference in a matrix of genetic network parameter estimates.

Hypothesis tests for networks have a variety of obvious applications. A one-sample comparison can occur when comparing a network estimate to a known ‘gold standard’ model. The standard could reside in an online ontology, be defined via a data processing algorithm (e.g., RMA normalization), or reflect the starting t_0_ state of a signal transduction network. Algorithms that derive networks using *in silico* or *in vitro/vivo* data could be tested for the comparability of their network model estimates in either a one- or two-sample context. For obvious reasons, two-sample comparisons have a broad application range. Two-sample tests are conducted by drug developers to compare pathways between an investigative compound at various doses or to a competitor’s compound. We conduct one- and two-sample tests using both simulated and microarray-based network data. To evaluate both Type I error control and the power of our procedure, we examine both null and non-null cases under a small set of network models. We assume that each sampling unit is an independent realization of a network. Our testing approach follows the traditional hypothesis testing route: define the null and alternate hypotheses (e.g., *η=η*_*0*_ versus *η>η*_*0*_ or *η*_*1*_*=η*_*2*_ versus *η*_*1*_*≠η*_*2*_) and the risk associated with a decision; define a ‘nearby neighbour’ dissimilarity-based test statistic for testing the hypothesis; compute the test statistic; compute the distribution of the test statistic under the null hypothesis; and, make a decision using the sampling distribution and the calculated test statistic. Our approach applies to edge/no-edge graphs, weighted graphs, and can be extended to directional networks. In the one-sample hypothesis testing context a null network model is assumed or based on resamples from an a priori null sample to generate the sampling distribution of our test statistic under the null hypothesis. To implement a two-sample comparison we rely on the permutation testing principle as the formal basis of our inferential approach [32].

## Methods

### Differential testing via dissimilarity

We consider an observed network as a realization of a stochastic process. A sample {*x*_*i*_*, i = 1, …, n*} of independent observations is therefore a set of networks. In contrast to social networks, where the sampling unit is often the node, we assume that the biological network is inherent to each sample observation. We propose a dissimilarity measure as a test statistic to capture the separation between two networks. We assume that we can align the nodes across the networks to be compared. Since each node in the network represents a gene or other molecular entity, the ability to align nodes across a set of networks implies that we know a unique identifier for each node to allow for a molecule-by-molecule comparison across a set of networks. We determine the sampling distribution of the test statistic under the null hypothesis via resampling techniques.

First, we begin with some definitions. Our definitions are consistent with Bollobás [7]. A graph *G* is an ordered pair of disjoint sets *(V,E)* where both *V* and *E* are finite sets. *V = V(G)* is the set of vertices and *E = E(G)* is the set of edges. An edge *{x,y}* is said to join, or tie, the vertices *x* and *y* and is denoted *xy*. If *xy* ∈ *E(G)* then *x* and *y* are adjacent, or neighbouring, vertices of *G* and the vertices *x* and *y* are incident with the edge *xy*. Two edges are adjacent if they have exactly one common endvertex. *G’ = (V’,E’)* is a subgraph of *G = (V,E)* if *V’ ⊂ V* and *E’ ⊂ E*. If *x* is a vertex of *G* we write *x* ∈ *G* in place of *x* ∈ *V(G)*. The order of *G* is the number of vertices in *G*; the size of *G* is the number of edges of *G*. *G(n,m)* denotes an arbitrary graph of order *n* and size *m*. A graph of order *n* and size

$$\left(\begin{array}{c} n\\ 2 \end{array}\right)$$

is a complete *n*-graph. A covariance matrix of nonzero elements with dimension *n, Σ*_*n*_, is a complete *n*-graph. The set of vertices adjacent to *x* ∈ *G,* the neighbourhood of *x*, is *Γ(x)*. For adjacent vertices *x* and *y* we have *x* ∈ *Г(y)* and *y* ∈ *Г(x)*. The degree of *x* is *|Γ(x)|*. A vertex of degree 0 is an isolated vertex (or isolate). A path is a graph *P* where *V(P) = {x*_*0*_*, x*_*1*_*, …, x*_*l*_*}* and *E(P) = {x*_*0*_*x*_*1*_*, x*_*1*_*x*_*2*_*, …, x*_*l-1*_*x*_*l*_*}*. The length of *P* is the size of *P*. A graph without any cycles, a path with length greater than or equal to three and comprised of distinct vertices, is an acylic graph. Unlike trees, we allow for cyclic graphs with isolates. Paths have an obvious tie to motifs and other regulatory functions. By definition, a loop (*xx* ∈ *E(G)*) is not allowed; multiple edges joining the same two vertices are not allowed. A graph *G* can contain a subgraph *G’* that is not connected to the remainder of *G*. Isolated nodes and subgraphs occur in biological networks. Accommodating isolates is necessary to align nodes between two graphs.

It is common to represent a graph *G* in matrix form. The adjacency matrix *A = A(G) = (a*_*ij*_*)* of a graph *G* is the *nxn* matrix given by

$$a_{\mathrm{ij}}=\left\{\begin{array}{c} 1,\;v_{i}v_{j}\in E\left(G\right)\\ 0,\;\text{otherwise}. \end{array}\right)$$

To extend the definition to a weighted graph replace 1 with *w*_*ij*_, where *w*_*ij*_ is the strength, covariance, etc., between vertices *v*_*i*_ and *v*_*j*_. Given *nxn* network matrices *G = (g*_*ij*_*)* and *H = (h*_*ij*_*)* we define *G-H* in the standard algebraic sense, i.e., *g*_*ij*_*-h*_*ij*_. We do not require a matrix be square; some directed network forms are *nxm* matrices. But, our use of element-wise subtraction is key; we are not suggesting a definition based on subspaces/subgraphs or set complements. We need to map an *nxn* network onto the real line in order to define a measure of separation. Under this definition of matrix subtraction, *G-H = 0*, where *0* is an appropriately dimensioned matrix of zeros, implies no separation between two networks.

The concept of dissimilarity (or similarity) is standard fare, especially in cluster analysis and pattern recognition. The dissimilarity measure *d*^*rs*^ between *r* and *s* satisfies the following: *d*^*rs*^*≥ 0* for every *r, s, d*^*rr*^*= 0* for every *r*, and *d*^*rs*^*= d*^*sr*^ for every *r, s*. Refer to Gan *et al*. [33] for an excellent catalogue of measures. Dissimilarity measures for categorical data *x* and *y* are generally based on a simple matching distance,

$$\delta \left(x,y\right)=\left\{\begin{array}{c} 0,\;x=y\\ 1,\;x\neq y \end{array}\right)$$.

The well-known Hamming distance [34] is a symmetrical form of a simple matching distance for binary strings and is used in communication theory. To craft our dissimilarity measure we propose a modified element-wise version of a matrix norm. Element-wise measures do not account for the interrelations present between nodes. Similar to linkage measures in genetics (e.g., LOD score), where markers are often correlated, we desire a measure that incorporates these interrelationships. Since networks are defined using interrelationships the inclusion of information for both a node and its neighbours is an intuitive concept.

Let *W*^*O*^*= (w*_*ij*_^*O*^*)* be a (weighted) adjacency (or directed incidence) matrix for the observed network estimate and *W*^*T*^*= (w*_*ij*_^*T*^*)* the target network. In a one-sample comparison *W*^*T*^ represents the true network model. For a two-sample comparison the distinction between the two labels is arbitrary. Both *W*^*O*^ and *W*^*T*^ represent graphs of order *n*; the nodes are labeled, common to, and aligned between both graphs. For node *i* define the dissimilarity at that node to be a combination of that node’s dissimilarity,

$$d_{i}^{\mathrm{OT}}=\overset{n}{\underset{j\neq i}{\sum }}\left|I\left(w_{\mathrm{ij}}^{O}\neq 0\right)-I\left(w_{\mathrm{ij}}^{T}\neq 0\right)\right|+\left|w_{\mathrm{ij}}^{O}-w_{\mathrm{ij}}^{T}\right|$$,

and the dissimilarity for node *i*’s neighbours,

$$d_{\mathrm{ij}}^{\mathrm{OT}*}=\overset{n}{\underset{k\neq i,j}{\sum }}\left|I\left(w_{\mathrm{jk}}^{O}\neq 0\right)-I\left(w_{\mathrm{jk}}^{T}\neq 0\right)\right|+\left|w_{\mathrm{jk}}^{O}-w_{\mathrm{jk}}^{T}\right|$$,

for nodes *j ≠ i, j* ∈ *Г(i)*. For the overall network, the dissimilarity *D* is defined as

$$D=\overset{n}{\underset{i}{\sum }}\left\{d_{i}^{\mathrm{OT}}+\overset{n}{\underset{j\neq i}{\sum }}d_{\mathrm{ij}}^{\mathrm{OT}*}c_{\mathrm{ij}}\right\}$$,

where

$$c_{\mathrm{ij}}=\left|w_{\mathrm{ij}}^{O}\right|I\left(w_{\mathrm{ij}}^{T}\neq 0\right)$$

for weighted networks and specified by the researcher for unweighted networks. *I* is defined as the standard mathematical indicator function. For a graph of order *n* a set/neighbourhood is formed at each node *w*_*ii*_*, i = 1,…,n*. We measure the dissimilarity between a node and its adjacent neighbours between the two graphs. To account for the intrinsic network structure the neighbourhood is then extended to those neighbouring nodes that are incident to nodes in *Γ(w*_*ii*_*)* for both the target and observed networks. I.e., we now measure the dissimilarity for the two subgraphs induced by *Γ(w*_*jj*_*)*, where *i ≠ j* and *w*_*jj*_ is an element of *Γ(w*_*ii*_*)*. This ‘extended’ neighbourhood dissimilarity is added to the dissimilarity measured at *w*_*ii*_. The contribution of the second nearest neighbours is weighted by *c*_*ij*_ in the definition of *D*. In a weighted network, e.g., a correlation network, this weight is easily motivated. In an unweighted network *c*_*ij*_ is set by the researcher. Assuming a weight of *c*_*ij*_*= 0* for an unweighted 0–1 graph reduces *D* to an unscaled version of the familiar Hamming distance and is comparable to the edit distance approach listed in [30].

We form *D* using separate edge and weight L_1_-norms. We elaborate on this choice later. The need to align the two graphs is critical to calculating *D*. Our approach does result in additional computational overhead since edge *xy* will be counted for nodes *x* and *y*. But, the counting is consistent and avoids the need for complex single-count network partitioning schemes. Only those nodes with a path length of 2 or less from *w*_*ii*_ are included; *D* can easily be extended to include path lengths greater than 2.

### One- and two-sample differential network comparisons

Defining an appropriate hypothesis in the context of networks can be nontrivial. For an Erdős-Rényi graph of order *n*, *G(n,p)*, the obvious parameter to test is *p*. Apart from ERGMs and (partial) correlation networks, explicit network parametric models may not be readily apparent. The basic form of a one-sample network hypothesis test here is *H*_*0*_*: η = η*_*0*_ versus *H*_*1*_*: η ≠ η*_*0*_, where *η* is anticipated to be a vector-valued parameter for most realistic (weighted) biological networks. For a *G(n,p)* graph we have *η = p* and one could test *H*_*0*_*:G(n,p) = G(n,p*_*0*_*)* versus *H*_*1*_*:G(n,p) ≠ G(n,p*_*0*_*)*. We intentionally provide no explicit guidance for how to determine *p*. And, we make explicit the probability model for the graph rather than state the hypothesis in a more compact manner, i.e., *H*_*0*_*:p = p*_*0*_. For a network defined using a correlation matrix *Ω* we construct hypotheses of the form *H*_*0*_*:Ω = Ω*_*0*_ versus *H*_*1*_*:Ω ≠ Ω*_*0*_. One needs to make explicit the procedure used to establish the edges in the network and the probability model for the observation data. In an example we test *H*_*0*_*:G(n,p) = G(n,p*_*0*_*)* versus *H*_*1*_*:G(n,p) > G(n,p*_*0*_*)*. Here, one needs to recognize that the amount of randomness/entropy for a *G(n,p)* graph is less when *p* is close to 0 or 1 relative to *p* close to 0.5 to successfully perform a one-sided test. Primarily, we are interested in the question, “Does this differ from the target?”

We employ a resampling approach to perform one- and two-sample network hypothesis tests. Following the five-step procedure outlined in Good [35] we first analyze the problem. We identify the null and alternate hypotheses under an assumed probability model and choose a suitable Type I error rate. Second, we select a test statistic to test the hypothesis. Here, *D* may be suitable in its stock form or require customization for the problem at hand. Third, compute the test statistic. Fourth, determine the distribution of the test statistic under the null hypothesis via a suitable resampling procedure. Finally, make a decision using the sampling distribution of the test statistics as a guide.

To generate a null distribution for *D* in the one-sample case we assume a parametric model or explicit generative algorithm is available in order to draw samples from the null network model. In most customary testing situations a test statistic is an estimate for a parameter of interest. However, in some cases the sample itself is the statistic – concise reductions of the data may be limited or not obvious/possible. Biological networks, where each edge or weight may be associated with transcription activity or a regulatory cascade, are inherently high-dimensional objects and may therefore lack parsimonious model parameterizations. In many applications a network algorithm *F* is required to produce an observed network. In these instances we may need to resample from *F(x)* instead of resampling from the observed data *{x*_*i*_*}*. In other cases, e.g., the Erdős-Rényi *G(n,p)* graph, the role of the *x*_*i*_ may be suppressed or not apparent since we observe *F(x)*. For networks based on explicit probability models, e.g., (partial) correlation networks, parametric bootstraps or Monte Carlo procedures may be possible under suitable assumptions [35].

Although a one-sample comparison has application for biomedical researchers, relative comparisons are of broad practical relevance. Research clinicians and pharmacologists are interested in exploring standard-of-care and new treatment comparisons for therapeutics. Transitioning to the *H*_*0*_*: η*_*1*_*= η*_*2*_ versus *H*_*1*_*: η*_*1*_*≠ η*_*2*_ two-sample problem allows one to draw upon established parametric and nonparametric comparisons. A null hypothesis of network equality versus an alternate hypothesis of network inequality is expected to be commonplace. Alternate hypotheses such as *H*_*1*_*: η*_*1*_*> η*_*2*_ are possible but not explored here. We make the standard assumption of two independent and identically distributed samples *{x*_*1*_*, …, x*_*n*_*}* and *{y*_*1*_*, …, y*_*m*_*}*, where *x*_*i*_ and *y*_*j*_ are network-valued. Following the notation of [35], let *P* be a family of distributions for *{X*_*1*_*, …, X*_*n*_*}* that are symmetric in the sense that for a permutation *π* of the subscripts *{1, …, n}* we have *P{( X*_*1*_*, …, X*_*n*_*)* ∈ *B} = P{( X*_*π(1)*_*, …, X*_*π(n)*_*)* ∈ *B}* for all Borel sets *B*. The random variables *X*_*i*_ are said to be exchangeable – a condition established under the assumption of independent and identically distributed samples or via the principle of randomization/random allocation in experimental design. As noted in Good, permutation tests rely on the assumption of exchangeability under the null hypothesis.

Permutation testing, a procedure which relies on samples drawn from the combined pool of experimental units and the random assignment of a treatment label to each unit, is common in the bioinformatics literature due to the prevalence of ‘*n<p*’ wide data and the lack of closed-form sampling distributions for various test statistics proposed. Pesarin [32] lists a variety of settings where these conditional inference procedures are useful. Some of the items listed that may apply to biological networks are: the distributional models for the responses are nonparametric, distributional models are not well-specified or may rely on too many nuisance parameters, the asymptotic null sampling distribution is unknown or depends on unknown quantities, or the sample size is less than the number of responses. To continue, these procedures might prove useful for multivariate problems where some variables are categorical (e.g., edge) and others quantitative (e.g., weight), in select multivariate inference problems where the component variables have different degrees of importance (e.g., edges discrepancies may be more severe than weight differences), and when treatment effects impact more than one aspect of the network. Applying the permutation testing principle, as stated in [32], to the two-sample network comparison problem via the customary mechanics serves as the inferential foundation for our two-sample testing strategy.

### Computer simulation

We first demonstrate *D* using an Erdős-Rényi *G(n,p)* graph. We test *H*_*0*_*:G(n,p) = G(n,p*_*0*_*)* versus *H*_*1*_*:G(n,p) > G(n,p*_*0*_*)*. The order of *G* is 25 and *p*_*0*_ is 0.20. We simulate four cases with 100 hypothesis tests (or experiments) in each case. For the null case we assume that the observed network is a *p = p*_*0*_ = 0.20 model. For the remaining two cases we assume that *p* = 0.25. We set *c*_*ij*_ = 0 for both a null and an alternate case and *c*_*ij*_ = exp(−2) for the remaining two cases. These four cases illustrate the Type I and II error rates of our procedure both with and without the inclusion of the neighbouring information in calculating *D*. 1,000 resamples were used to estimate the null distribution for *D*. The execution time for the set of 100 experiments using 1,000 resamples was approximately 1 hour on a standard personal computer. The R package Statnet [36] was used to generate the *G(n,p)* graphs. All of our computations were conducted using R (http://www.r-project.org/). For testing large networks or large numbers of networks a more computationally efficient language such as Fortran or C++ is recommended. The representation diversity and size of networks, combined with a need or interest to tailor *D* for a particular application, poses a challenge for software developers.

To evaluate *D* for a correlation-based network model we assume that the *p*-dimensional observations follow a multivariate normal distribution, *N*_*p*_*(μ,Σ)*, with mean vector *μ* and positive definite covariance matrix *Σ*. Transforming *Σ* into correlation form *Ω* allows us to form a partial correlation matrix. Applying a threshold to each *ρ* estimate or a testing procedure to the entries of *Ω* can be used to define a correlation network. Given *Ω*^*-1*^*= (ω*_*ij*_*)* we compute the partial correlation matrix *Π = (π*_*ij*_*)* via *(π*_*ij*_*) = −ω*_*ij*_*/√(ω*_*ii*_*ω*_*jj*_*)*. Under multivariate normality two variables are conditionally independent given the remaining variables if and only if the partial correlation vanishes. The zeros in *Ω*^*-1*^ determine the conditional independence graph. As before, a threshold or testing procedure is used to define the partial correlation network. We evaluate both the Type I error rate and the power of *D* under an alternate hypothesis.

Apart from the Erdős-Rényi example, the one- and two-sample simulation comparisons assume that the observations are in their correlation form, i.e., *x*_*i*_*~ N(0, Ω).* To mimic a sparse biological network *Ω* consists of 6 nonzero 5x5 blocks along the diagonal. A sample rejection scheme guaranteed that the magnitude of each block entry exceeded a predefined threshold. The number of nodes (30) and the threshold (*ρ* = 0.2) was common to all simulations. The same threshold *ρ* was used to define the data generation model and to estimate the observed correlation network in the one-sample case. For both comparisons we evaluated *D* using sample sizes of *n*_*1*_*= n*_*2*_ = 200. For the one-sample comparison the target correlation network *Ω*_*0*_ is estimated from the sample data and the resamples are drawn from the observed samples to determine the null distribution for *D*. Admittedly, this approach violates the true spirit of a one-sample test under an assumed null model. But, if historical sample observations are available for analysis and reflect the null hypothesis then these samples might provide the most scientifically defensible null distribution for *Ω*_*0*_. The same approach is used for the derived microarray-based correlation network comparison; a case where the true null model is unknown. To simulate the alternate hypothesis of network inequality at least 10% of the 5 × 5 blocks for a 30 × 30 correlation matrix, with a minimum of one block per experiment, were varied using a random number generator. A total of 100 experiments were performed and 1,000 resamples used in calculating each p-value. For both the correlation and partial correlation networks we calculate *D* using only the weight portion of the index since the existence of an edge was defined via the (partial) correlation estimate. The execution time for the 100 experiments using 1,000 resamples was approximately 1–2 hours on a standard personal computer for the one-sample comparison. The two-sample setting execution time was on the order of 4–6 hours. For select simulation data the algorithm used to estimate the partial correlation network, see below, would abruptly terminate. All of the resample p-values shown here were obtained upon a successful completion of the estimation process. The computation time for the actual biological data was negligible. This is likely due to the small networks involved; network comparisons involving a large number of nodes and/or edges present a non-trivial computational burden. Refer to Additional file 1 for the R code used.

We selected partial correlation networks as presented in [37] (commonly referred to as Gaussian graphical models, or GGMs, for multivariate normal observations) for the two-sample comparisons due to their use in the literature, e.g., De la Fuente *et al*. [38] use partial correlations up to order 2 to model genomic data, and partial correlations are formed using a plurality of variables – an intuitive appeal for defining a network. Markowetz *et al*. [39] suggest that partial correlations may better reflect interdependencies in a network relative to a standard correlation coefficient. We fit these networks using the GeneNet algorithm presented in Opgen-Rhein *et al*. [40], an extension of the algorithm from Schäfer *et al*. [37]. The GeneNet R package is available from the CRAN R archive (http://cran.r-project.org). The algorithm broadly consists of 3 steps. First, the correlation (or covariance) matrix *Ω* is converted to a partial correlation matrix *Π*; pseudoinverses may be involved. The second step tests for the presence of ‘significant’ edges. The final step extracts the significant edges based on a user-defined criterion. We used the magnitude of the estimated (shrunken) partial coefficient to establish this cut-off – the cutoff.ggm parameter for *π*_*ij*_ was set to 0.5. Default settings were used for the remainder of the GeneNet settings. We do not claim that GGMs are superior for modeling gene/protein networks. [37] demonstrate via simulation that the quality of various partial correlation estimators can vary according to the sample size and dimensionality of the observed data. [39] document the need for larger samples in the practical use of GGMs.

### Type II diabetes mellitus data: one-sample comparison

Type II diabetes mellitus (DM2) is a serious metabolic disorder affecting a large number of people worldwide. Mootha *et al*. [41], using microarray transcriptional profiles obtained from 17 normal and 17 DM2 muscle biopsy samples, presented a study in conjunction with the Gene Set Enrichment Analysis (GSEA) tool to detect differential expression patterns among functionally-related gene sets. They identified a single gene set, OXPHOS – an oxidative phosphorylation pathway, which exhibited differential gene expression levels between the two phenotypes and linked this gene set to clinically important variation in human metabolism. Mootha *et al*. analyzed 149 gene sets – 113 were selected based on their involvement in metabolic pathways and the remainder based on co-regulated gene clusters from a mouse expression atlas. The transcript expression data and the gene set definitions from the original GSEA study were obtained from the authors’ website [42].

The original study examined average expression levels across the two groups. Here, we explore the differences in covariance or correlation structures between the two phenotypes as expressed in a correlation network formed via a threshold for *ρ*. To facilitate a one-sample comparison the normal tissue samples are used to define a target network. The resampling procedure was outlined in the previous section. Rather than analyze severely ill-conditioned correlation matrices (numerous gene sets contained over 100 genes), we formed correlation networks only for those gene sets with fewer than 18 probes in the pathway. A significant one-sample finding for one or more gene sets may suggest differences in co-regulation network behaviour of a person with DM2 relative to a normal subject.

### Ovarian cancer: two-sample comparison

Ovarian cancer is the foremost lethal neoplasm of the female genital tract. We examine here the gene expression signatures of ovarian serous carcinomas (SCAs) relative to serous borderline tumors (SBTs) based on three recent studies. Sieben *et al*. [43] confirmed the activated role of a mitogenic pathway in SBTs and uncovered downstream genes that helped differentiate SBTs and SCAs. De Meyer *et al*. [44] investigated the role of the *E2F/Rb* pathway in SBTs and SCAs. Chien *et al*. [45] demonstrated the significance of the *p53* and *E2F* pathways in serous carcinomas and reinforced the role of *E2F*s listed in [44]. These three studies demonstrate that differential expression patterns exist between SBTs and SCAs. We examine a subset of these data to test whether or not covariation patterns differ between SBTs and SCA1s (low-grade carcinomas) and between SCA1s and SCA3s (high-grade carcinomas). Changes in covariation patterns may assist in the design of follow-up studies, suggest a novel biomarker test or a better categorization of SCAs, or provide insight into a patient’s responsiveness to chemotherapy agents.

The microarray data analyzed here was obtained from the NCBI GEO database [46] via accession number GSE12471. These data were originally presented in [43] and contain the mRNA expression profiles of 11 SBT, 10 SCA1, and 15 SCA3 samples. 2 micropapillary pattern SBT samples were omitted from our analysis. De Meyer *et al*. [44] screened the original expression profiles to reduce the number of genes examined and cross-referenced their *E2F* target genes with the studies of Bracken *et al*. [47] and Bieda *et al*. [48]. 43 of these genes were classified by 6 biological processes in [47]: 5 for the G1/S phase of the cell cycle, 13 from the S/G2 phase of the cell cycle, 6 checkpoint genes (e.g., *BRCA2*), 1 development gene, 5 DNA damage and repair genes, and 13 DNA synthesis/replication genes. A table of the specific genes included is in Additional file 2. Excluding the singleton subset, we estimated partial correlation networks for each of the 5 process-defined gene subsets across the three carcinoma subtypes.

## Results

### Simulation study

**Figure 1:**
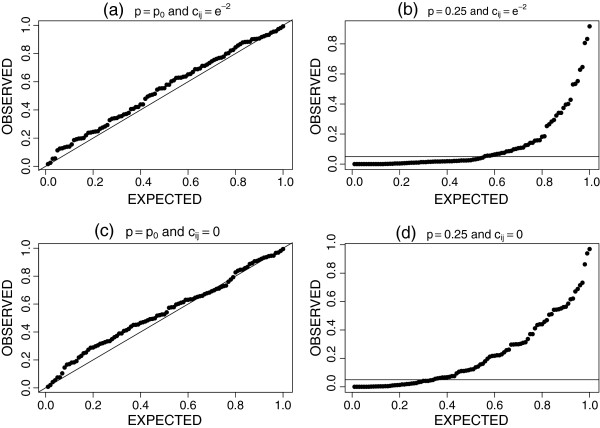
**One-sample tests for an Erdős-Rényi graph.** P-value results from 100 independent tests of *H*_*0*_*:G*(25,*p*) = *G*(25,0.20) versus *H*_*1*_*:G*(25,*p*) > G(25,0.20). The y-axis is the observed resample p-value; the x-axis is the expected p-value under the null hypothesis. Panels (**a**) and (**c**), via a uniform distribution qq-plot, illustrate the Type I error rate using 2 settings for *c*_*ij*_. Panels (**b**) and (**d**) illustrate the performance of *D* under the alternate hypothesis for 2 settings of *c*_*ij*_; a horizontal line corresponding to an α = 0.05 level is provided.

For the Erdős-Rényi *G*(*n*=25, *p*=0.20) random graph comparison refer to Figure 1. Apart from the appearance of a weak bias for both the *c*_*ij*_ = 0 and *c*_*ij*_ = exp(−2) cases the p-values are approximately uniformly distributed under the null hypothesis. In panels (a) and (c) we see that the nominal Type I α level is approximately 0.05. Comparing panel (b) to (d) we see the improvement in the power of *D* when *p* = 0.25 and *c*_*ij*_ = exp(−2). When the neighbouring information was not used in calculating *D* 34% of the resample p-values were below a nominal α level of 0.05. When the neighbouring information was included in *D* and scaled by *c*_*ij*_ = exp(−2) 55% of the p-values were below the nominal 0.05 level. The likelihood of *D* detecting the true alternative hypothesis increased from 34% to 55% just by including the neighbouring information here.

**Figure 2:**
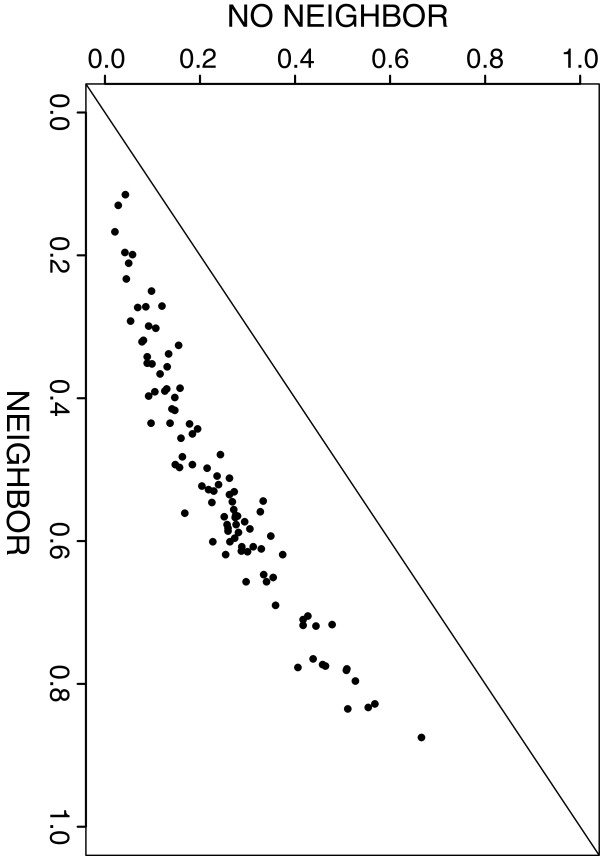
**One-sample comparison for a correlation network under H**_**1**_**.** 100 resample p-value for a test of *H*_*0*_*: Ω = Ω*_*0*_ versus *H*_*1*_*: Ω ≠ Ω*_*0*_ under an assumed alternate hypothesis. The y-axis indicates the p-value obtained excluding the use of the neighbouring information in calculating *D*; the x-axis corresponds to the p-value obtained using the neighbouring information in calculating *D*.

For the one-sample correlation network comparison we examined both the Type I error rate and the power of *D* to reject *H*_*0*_*: Ω = Ω*_*0*_ versus *H*_*1*_*: Ω ≠ Ω*_*0*_. The tests were performed using *c*_*ij*_ = 0, i.e., excluding the neighbouring information, and *c*_*ij*_*= r*_*ij*_, the thresholded correlation estimate. Under the assumed null model the distribution of p-values, for both *c*_*ij*_ = 0 and *c*_*ij*_*= r*_*ij*_, had less mass at the extremes of the p-value range. Additional simulation work, see [49], suggested that when *n*_*1*_*= n*_*2*_ = 100 the Type I error rate was conservative, produced an inflated error rate when *n*_*1*_*= n*_*2*_ = 2,000, and the test achieved a proper size when the a priori sample size was a factor of 10 larger than the observed network based on a sample of *n* = 200. These results suggest caution when trying to determine the null distribution for *D* using a finite set of a priori samples. Figure 2 graphs pairs of p-values obtained from 100 experiments under the alternate hypothesis. This figure demonstrates that incorporating the neighbouring information in the calculation of *D* via *c*_*ij*_*= r*_*ij*_ penalized our ability to reject the null hypothesis when compared to the use of *c*_*ij*_ = 0.

**Figure 3:**
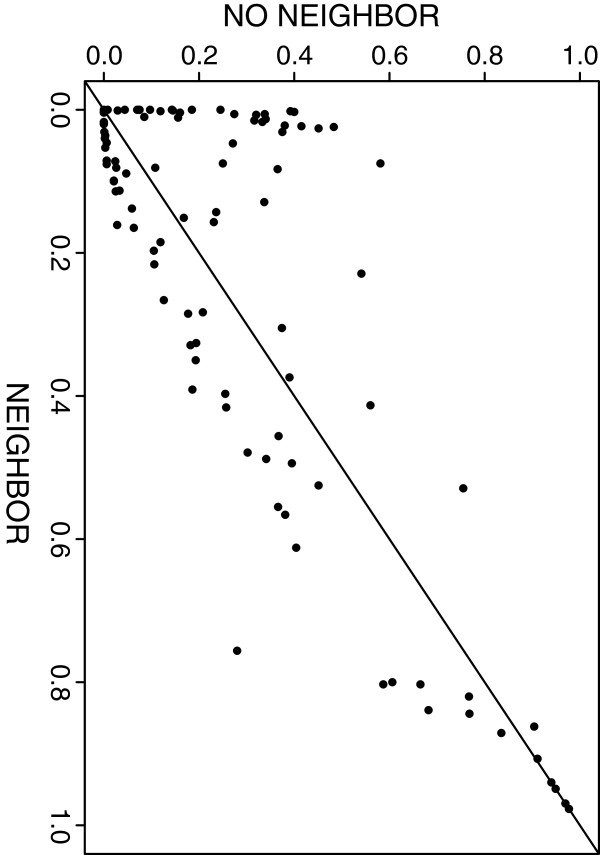
**Two-sample comparison for partial correlation networks under H**_**1**_**.** 100 resample p-value for a test of *H*_*0*_*: Π*_*1*_*= Π*_*2*_ versus *H*_*1*_*: Π*_*1*_*≠ Π*_*2*_ under an assumed alternate hypothesis. The y-axis indicates the p-value obtained excluding the use of the neighbouring information in calculating *D*; the x-axis corresponds to the p-value obtained using the neighbouring information in calculating *D*.

Additional file 3 graphs the Type I error performance for the two-sample comparison under the null hypothesis of partial correlation network equality, *H*_*0*_*: Π*_*1*_*= Π*_*2*_. In Figure 3 we plot the pairs of p-values obtained under an alternate hypothesis. In both figures we illustrate the inclusion/exclusion of the neighbouring information in calculating *D* (i.e.,

$$c_{\mathrm{ij}}=0\;\mathrm{or}\;c_{\mathrm{ij}}={\hat{\pi }}_{\mathrm{ij}}$$).

Under the null hypothesis we see that the p-values are approximately uniformly distributed with reasonable Type I error control. Including the neighbouring information does suggest more lack of fit; this is not surprising given the data’s correlated block structure and the correlated components used in *D*. Under the alternate hypothesis simulated here 45 of the p-values determined with the neighbouring information were less than the corresponding p-value calculated excluding the neighbouring information, a result comparable to the flip of a fair coin. The more dramatic result is comparing the number of times we reject *H*_*0*_ at an alpha level of 0.05. Under *H*_*1*_, 40 of the p-values were less than 0.05 when *D* included the neighbouring information; 24 of the p-values were less than 0.05 when *D* excluded the neighbouring information. This gain in power by including the neighbouring information differs from the one-sample correlation network simulations. But, as the p-values shift away from 0, and move in favour of *H*_*0*_, excluding the neighbouring information consistently produced smaller p-values. This suggests the behaviour of *D* may vary according to the network inference method employed.

### Comparison of derived biological networks

We first compare the correlation networks for the DM2 phenotype to the Normal phenotype. We test *H*_*0*_*: Ω*_DM2_ = *Ω*_Normal_ versus *H*_*1*_*: Ω*_DM2_ ≠ *Ω*_Normal_ where we assume *Ω*_Normal_ is known. We form Pearson product-moment-based correlation networks only for those gene sets with fewer than 18 probes in the pathway. The 17 normal samples were used to form *Ω*_Normal_. We emphasize here the potential power of our test since the true state of the null or alternate hypothesis is unknown. To generate the null distribution for *D* we resample, with replacement, from the original 17 normal samples. 1,000 resamples were used throughout. As in the simulation study, the level of the test may be unreliable due to the resampling procedure employed. Estimating an overparameterized correlation matrix based on small samples is a general challenge for the practical estimation of network models.

**Figure 4:**
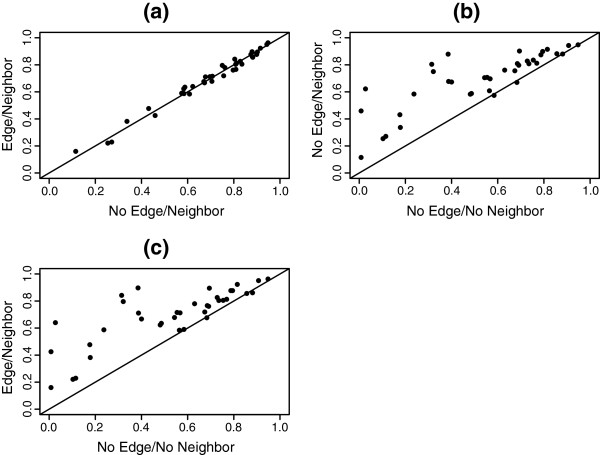
**One-sample correlation network comparison of Type II diabetes versus Normal phenotype.** Resample p-values for the 37 gene sets analyzed. Edge/no-edge indicates the inclusion/exclusion of the edge portion in calculating *D*. Neighbour/no-neighbour indicates the inclusion/exclusion of the neighbouring information in calculating *D*.

Comparable to the earlier simulation study Figure 4 illustrates the performance of *D* including/excluding the edge portion of *D* and including/excluding the neighbouring information at a correlation threshold of *ρ* = 0.5. Panel (a) suggests that the edge portion of *D* is redundant to the weight component and panels (b-c) suggest that including the neighbouring information detracts from the power of *D*. Table 1 lists the resample p-values for the 37 network comparisons performed at correlation thresholds *ρ* = 0.35 and *ρ* = 0.5 (see [49] for additional results). The edge portion was excluded from *D* and, despite the potential loss in power, the neighbouring information was included. Even at less conservative Type I error levels we fail to declare a network difference in the majority of the cases. The p-values have not been adjusted for the number of comparisons. To illustrate the estimated correlation networks for the two phenotypes near the p-value extremes we provide the Normal and DM2 correlation network estimates for the MAP290 and MAP472 gene sets in Table 2. Yates [49] provides a post hoc analysis of the MAP290 correlation network under the assumption of a declared significant network difference.

**Table 1:** One-sample correlation network comparison of Type II diabetes versus Normal phenotype

**Gene Set Name**	***ρ*=0.35**	***ρ*=0.50**
1 KET-HG-U133A probes	0.38	0.828
2 MAP31 Inositol metabolism	0.607	0.608
3 MAP40 Pentose&glucuronate interconversions	0.599	0.574
4 MAP53 Ascorbate&aldarate metabolism	0.455	0.809
5 MAP62 Fatty acid biosynthesis path 2	0.644	0.761
6 MAP72 Synthesis&degradation of ketone bodies	0.588	0.915
7 MAP130 Ubiquinone biosynthesis	0.122	0.115
8 MAP140 C21 Steroid hormone metabolism	0.901	0.902
9 MAP271 Methionine metabolism	0.49	0.879
10 MAP272 Cysteine metabolism	0.522	0.584
11 MAP290 Valine leucine&isoleucine biosynthesis	0.139	0.431
12 MAP400 Phenylalanine tyrosine&tryptophan biosyn	0.443	0.804
13 MAP430 Taurine&hypotaurine metabolism	0.782	0.705
14 MAP450 Selenoamino acid metabolism	0.554	0.874
15 MAP460 Cyanoamino acid metabolism	0.569	0.808
16 MAP472 D-Arginine&D-ornithine metabolism	0.916	0.948
17 MAP511 N-Glycan degradation	0.58	0.677
18 MAP512 O-Glycans biosynthesis	0.613	0.673
19 MAP522 Erythromycin biosynthesis	0.081	0.254
20 MAP532 Chondroitin Heparan sulfate biosynthesis	0.726	0.882
21 MAP533 Keratan sulfate biosynthesis	0.861	0.943
22 MAP580 Phospholipid degradation	0.484	0.271
23 MAP601 Blood group glycolipid biosyn lact series	0.571	0.588
24 MAP603 Globoside metabolism	0.92	0.88
25 MAP630 Glyoxylate&dicarboxylate metabolism	0.276	0.622
26 MAP631 1-2-Dichloroethane degradation	0.473	0.797
27 MAP632 Benzoate degradation	0.515	0.812
28 MAP680 Methane metabolism	0.319	0.337
29 MAP720 Reductive carboxylate cycle CO2 fixation	0.085	0.459
30 MAP740 Riboflavin metabolism	0.231	0.583
31 MAP760 Nicotinate&nicotinamide metabolism	0.581	0.899
32 MAP780 Biotin metabolism	0.451	0.67
33 MAP900 Terpenoid biosynthesis	0.802	0.835
34 MAP950 Alkaloid biosynthesis I	0.666	0.6965
35 MAP3030 DNA polymerase	0.877	0.707
36 PYR-HG-U133A probes	0.524	0.75
37 ROS-HG-U133A probes	0.658	0.756

Resample p-values for the 37 gene sets analyzed based on correlation networks using thresholds *ρ* = 0.35 and 0.50.

**Table 2:** Valine leucine and isoleucine biosynthesis (MAP290) and D-Arginine and D - Ornithine Metabolism (MAP472) gene sets for the Normal and Type II (DM2) phenotypes

**MAP 290**												
**Probe**	**Normal**						**DM2**					
1	1.0	.	.	.	.	.	1.0	.	.	.	.	.
2		1.0	0.53	0.59	0.59	.		1.0	.	.	.	.
3			1.0	0.73	.	.			1.0	0.81	.	.
4				1.0	0.86	.				1.0	.	.
5					1.0	.					1.0	.
6						1.0						1.0
**MAP 472**												
1	1.0	0.62	0.61	0.56	0.60	0.52	1.0	0.63	0.62	0.64	0.57	0.54
2		1.0	0.99	0.98	0.97	0.94		1.0	0.99	0.97	0.98	0.94
3			1.0	0.98	0.97	0.95			1.0	0.97	0.96	0.95
4				1.0	0.97	0.97				1.0	0.98	0.96
5					1.0	0.96					1.0	0.94
6						1.0						1.0

The numerical values are the thresholded correlation estimates. A ‘.’ denotes the lack of an edge between two probes, ‘1.0’ is a visual placeholder. The actual probes differ between the two gene sets.

For the two-sample comparisons we examined a limited ordered comparison of the SBT, SCA1, and SCA3 phenotypes for the 5 biological processes. Our subjective rationale was that a comparison of the SBT and SCA1 phenotypes might suggest a biomarker candidate; comparing the SCA1 and SCA3 phenotypes might better characterize disease progression or provide insight regarding resistance to chemotherapy agents. Table 3 lists the number of edges in the estimated partial correlation networks obtained using GeneNet for the subsets categorized by Bracken *et al*. [47]. Apart from the DNA synthesis and replication process the estimated GGMs are either empty or sparse. As a side note – some of our simulation work suggests that GeneNet tends to underfit a network. Table 4 lists the resample p-values for a test of *H*_*0*_*: Π*_*1*_*= Π*_*2*_ versus *H*_*1*_*: Π*_*1*_*≠ Π*_*2*_ across the eight comparisons. All of these comparisons used the neighbouring information in calculating *D*; apart from the smallest p-value listed the differences between the neighbour/no-neighbour p-values were negligible. At a standard alpha level of 0.05, a conservative value for a comparison of covariance-based matrices, none of the hypotheses would be rejected. Rather than adopt such a conservative view, and ignoring the topic of multiple comparisons, we chose to provide the partial correlation networks for the 13 genes in the SCA1 and SCA3 phenotypes (p-value 0.142) in Table 5. This table suggests an observable difference between the two networks.

**Table 3:** Gaussian graphical model estimate details for three ovarian cancer phenotypes

**Biological Process**	**No. of Genes in Process**	**SBT**	**SCA1**	**SCA3**
G1-S phase of the cell cycle	5	0	0	3
S-G2 phase of the cell cycle	13	0	2	0
Checkpoint	6	0	3	4
DNA damage and repair	5	4	0	0
DNA synthesis and replication	13	0	30	40

Number of edges in the Gaussian graphical model estimate for each of the three phenotypes across the five processes categorized by Bracken *et al*. [47].

**Table 4:** Two-sample comparison of select ovarian cancer phenotypes

**Biological Process**	**SBT versus SCA1**	**SCA1 versus SCA3**
G1-S phase of the cell cycle	NA	0.636
S-G2 phase of the cell cycle	0.676	0.691
Checkpoint	0.812	0.380
DNA damage and repair	0.637	NA
DNA synthesis and replication	0.368	0.142

Resample p-values for phenotypic comparisons of the form *H*_*0*_*: Π*_*1*_*= Π*_*2*_ versus *H*_*1*_*: Π*_*1*_*≠ Π*_*2*_.

**Table 5:** Estimated networks for the SCA1 and SCA3 phenotypes

**SCA1**													
*PCNA*	1.0	.	.	.	0.60	0.65	.	−0.75	.	−0.59	−0.59	.	.
*TOP2A*		1.0	−0.64	.	.	.	0.53	.	.	.	.	0.52	−0.48
*MCM3*			1.0	−0.43	.	−0.41	.	.	0.66	.	.	0.88	−0.72
*MCM6*				1.0	.	.	−0.72	.	0.64	.	.	0.49	−0.44
*MCM2*					1.0	.	.	.	−0.46	0.55	.	.	.
*TK1*						1.0	.	0.66	0.47	0.50	.	.	.
*CDC6*							1.0	0.58	.	.	.	.	.
*RFC4*								1.0	.	.	−0.77	.	.
*CDC45L*									1.0	.	.	−0.62	0.80
*RFC3*										1.0	.	.	.
*POLA2*											1.0	0.61	−0.43
*CDC7*												1.0	0.77
*RRM2*													1.0
**SCA3**													
*PCNA*	1.0	−0.52	.	.	0.37	0.42	0.64	.	0.34	.	.	0.34	−0.44
*TOP2A*		1.0	0.36	−0.49	0.37	.	0.84	.	0.62	0.35	.	.	−0.56
*MCM3*			1.0	.	0.47	.	.	0.47.	−0.40	−0.60	−0.40	.	.
*MCM6*				1.0	0.60	0.34	.	−0.40	.	0.37	.	−0.43	.
*MCM2*					1.0	−0.44	−0.43	.	.	.	.	0.45	.
*TK1*						1.0	−0.35	.	.	.	.	.	.
*CDC6*							1.0	.	−0.42	.	0.39	.	0.70
*RFC4*								1.0	0.34	0.82	0.49	−0.46	.
*CDC45L*									1.0	−0.37	.	.	0.60
*RFC3*										1.0	−0.58	0.34	.
*POLA2*											1.0	0.46	.
*CDC7*												1.0	.
*RRM2*													1.0

The estimated SCA1 and SCA3 Gaussian graphical model networks for the DNA synthesis and replication genes. Off-diagonal non-zero weights indicate the presence of an edge between two genes, ‘.’ denotes the lack of an edge, and ‘1.0’ is a visual placeholder.

## Discussion

The definition of *D* was inspired by the question, “How long is the coast of Britain?” This question motivates the definition of fractal dimension, a concept suited for complex objects. Cutler’s [50] definitions of packing and pointwise dimension suggest measures based on an additive decomposition of sets/neighbourhoods. The neighbourhood size and scaling behaviour can vary across the points in the set. Combining these ideas with a Riemann-like sum is our basis for a topological comparison of networks. Nacu *et al*. [51] suggest the use of neighbourhoods, of potentially varying radius, for identifying differentially expressed pathways. Choosing a node as the centre of the local neighbourhood, and not an edge, allows for several advantages: a reduction in combinatorial complexity, facilitates molecular node-based post hoc tests, avoids the need for an ‘optimal’ tiling or network partition, and it limits the number of relational features/motif-like structures to compare. We limited our set/neighbourhood-based definition of *D* to internodal path lengths strictly less than three since this is the minimum distance necessary to capture a feedforward/feedback loop, it limits the range of topological comparisons, and *D* is prevented from revisiting the ‘centre’ of a neighbourhood in a cyclic graph. *D* can be extended to include a larger neighbourhood. Apart from obvious computational implications, defining a second set of *c*_*ij*_’s is necessary and the efficiency of *D* may deteriorate as *D* integrates a larger set of imprecise or variable estimates. In contrast to fractals, viewing a network as an inhomogeneous mixture of subgraphs suggests that a local distance may be preferable to large distances that span a large set of nodes.

We do not claim that *D* is or will be optimal under a broad range of network models. Apart from the literature related to ERGMs, weighted biological network models with well-developed inferential theory analogous to maximum likelihood, uniformly minimum-variance unbiased, or minimax estimators is largely unavailable. The ability to apply *D* to a broad range of edge/no-edge and weighted/unweighted networks is sure to involve tradeoffs. Banks *et al*. [52] illustrate a case where a metric for a clustering application is ill-suited for a phylogenetic inference problem. [53] state the need for fixed/absent edges in their informative prior approach to network inference. A decomposable additive measure can be tailored to reflect meaningful comparisons, e.g., [54] modify an L_1_-based edit distance for unweighted binary networks using protein signaling logic. While not constituting a definitive argument, we speculate that the value of including the neighbouring information in the *G(n,p)* simulation example is due to the uniformity of *p* across the set of nodes. The loss in performance using the neighbouring information for a pairwise correlation network model is likely due to *D* unnecessarily integrating across a larger set of imprecise estimates. For the two-sample partial correlation network comparison, the gain in performance via the use of neighbours may be influenced by the fact that each edge is formed using a plurality of variables. For researchers interested in alternate network models, e.g., preferential attachment or small-world models, evaluating *D* (or comparing it to a competing procedure) under various scenarios is recommended.

Basing *D* on a more general form of a Hamming distance parallels efforts in the area of global network alignment scoring. Others have proposed alignment-based operations for use with biological networks, e.g., [55] for comparing phylogenetic trees. The use of set algebraic operations, e.g., union, intersection, strict and symmetric differences, is commonplace [20,21,56]. Having *D* incorporate neighbouring information might be obvious to a systems biologist; see [57] for an example in classifying breast cancer metastasis and [58] for a pathway differential expression application. [38], using partial correlations, found it useful to restrict the number of genes/nodes to condition on. [59] use level-1 and level-2 neighbours to predict protein function. [23] outline a weighted topological overlap measure to cluster gene modules. [22] present a topological overlap measure that generalizes pairwise similarity to one based on shared neighbours. The precedent of incorporating neighbouring information for network objects is clear; the question of, “But how far do we go?” is less clear. As mentioned earlier, the matter of neighbourhood size is liable to impact the behaviour of *D* and may interact with network estimation procedures, data collection requirements, etc.

Despite *D*’s simplicity and potential for use across a broad set of network models, the importance (and weighting) of *D*’s components should receive serious consideration by the investigator. Gower [60] concedes that weighting components of a measure is challenging. A robustness study is recommended to address this topic, see Yates [49]. For both one- and two-sample comparisons, tuning *D* to gage or improve its performance for a specific network model is advised. The selection of the weight constant *c*_*ij*_ for weighted graphs was motivated by the idea of conditional probabilities. If *A* and *B* represent two adjacent edges and information flows through their common vertex then it is reasonable to assume that some form regarding the state of *B* is meaningful to *A*. It is plausible to assume that the force two objects exert on one another is proportional to their proximity. Preferential attachment networks assume that new edges are formed at a node conditional on the number of existing edges at that node. Wei *et al*. [61] employ gene-specific prior probabilities in a spatially correlated mixture model application. But, the quality of the weights may be suspect. Ashyraliyev *et al*. [62] found quantitative parameter estimates to be unreliable in modeling a gap gene circuit but that inferring a reliable qualitative network topology was possible. The relative weighting of edge and weight differences is a topic to discuss here. The idea of scaling or normalizing portions of *D* is complicated. Gao *et al*. [63] allow hubs to exert an unequal influence; Yip *et al*. [64] normalize their generalized topological overlap measure to the unit interval. Given a rough similarity between *D* and the total sums of squares in regression modeling we allow for nodes with a high degree (i.e., ‘large degree of freedom’ tests) to contribute more to the calculation of *D*. If one chooses to normalize portions of *D* at each node by some topological property then one has to justify the scaling factor. Does one scale by the node’s (weighted) degree, a clustering coefficient, in- or out-degree for a directed network, etc.? If a network model has an efficient estimator whose sampling distribution is well characterized, the use of *D* may be contraindicated. At present, we do not expect this to be the case for experiment-based weighted biological networks. As a conservative measure, *D* could be reduced to its Hamming-like form and only the inferential procedures outlined here applied. Evaluating *D* using an *in silico* model under plausible scientific scenarios of interest to the investigator could also reduce concerns about aspects of the definition of *D*.

Our choice of network architectures to evaluate with *D* was limited. Computational expediency, an ability to explore and contrast common parametric models and their associated weighted network forms, and the use of a canonical structure (e.g., *G(n,p)* graphs) motivated our network model selection. Both Marbach *et al*. [65] and Altay *et al*. [66] discuss difficulties associated with currently available network inference algorithms. Müller-Linow *et al*. [67] found that the proximity of metabolites in a correlation network did not align with metabolite proximity observed in genome databases. Hubert *et al*. [68] suggest that final structural representations are unlikely to be global optima since the selection procedure, while reasonable, does not make use of a verifiable optimal search strategy. Even the limited simulation work presented here suggests the nuance that network algorithms or models can inject into the process. Relying on a priori samples or an assumed null model to generate a sampling distribution for *D* may be restrictive in the one-sample case; but, the complexities surrounding network probability models and the intractability of network-based test statistics does not suggest easy alternatives. Large (weighted) networks are likely to be costly in terms of the data needed to estimate a family of network parameters. The literature on the analysis of large networks, with its emphasis on select topological comparisons, typically imposes a vast reduction in network complexity. Thorne *et al*. [69], in proposing a method to generate confidence intervals for network-related correlations and motif-abundances reinforced the complexities in defining a suitable null model for a biological process.

General criticisms leveled against the use of resampling methods are applicable here; see Berger [70]. The practical matter of using *D* for observation studies raises the topic of partial exchangeability. It is unreasonable to assume that either of the biological datasets presented here met the assumption of exchangeability. However, unconditional procedures also struggle when confronted with observational data and/or missing or hidden covariates. Our one- and two-sample comparison approaches require or assume a hypothesized network model for the purpose of sampling; we do not employ fixed-network random rewiring schemes or variations that seek to align a set of topological measures across a set of stochastic networks.

A significant one- or two-sample finding naturally suggests the question, “Where do the networks differ?” Similar to individual effect tests in regression, the answer to this question will involve one or more nodes or subgraphs. Answering targeted questions routinely appear in the literature, see [71,72]. Dong and Horvath [73] suggest approximately factorizable networks; [74] locate regulatory hot-spots by integrating network topology and transcriptome data. Since *D* is a sum of node-based dissimilarities, a test for dissimilarity between two aligned nodes or subgraphs can be formed using the portion of *D* attributable to that node or subgraph. Such an approach may help isolate molecular targets or subnetworks for closer study. Determining the sampling distribution for the modified test statistic is identical to the procedure developed here. See Yates [49] for more details and examples.

## Conclusions

In this paper we have demonstrated an inferential framework for performing one- and two-sample hypothesis tests for biological networks. We proposed a suitable test statistic and evaluated it with both simulated and microarray data under a variety of situations. The dissimilarity test statistic proposed is a logical extension of existing measures used in sequence alignment/nominal data comparisons. In generating the null distribution of the test statistic we used an assumed parametric model, resamples from a collection of null samples, and made use of permutation testing principles. Resampling methods suggest that counterintuitive behaviours may occur when dealing with a (potentially complex) network model. While not explicitly demonstrated here, our procedure easily facilitates node-level or subgraph post hoc tests.

## Competing interests

The authors declare that they have no competing interests.

## Authors’ contributions

This work is based on the PhD dissertation of PDY. NDM suggested the problem and supervised the research. All authors read and approved the final manuscript.

## Supplementary Material

Additional file 1: R code. Click here for file

Additional file 2: **Ovarian cancer genes analyzed.** Subset of analyzed genes as categorized by Bracken *et al*. [47]. Click here for file

Additional file 3: **Two-sample comparison for partial correlation networks under H**_**0.**_ A uniform qq-plot of the 100 resample p-values for a test of *H*_*0*_* Π*_*1*_*= Π*_*2*_ versus *H*_*1*_* Π*_*1*_*≠ Π*_*2*_ under the null hypothesis. The y-axis is the observed p-value; the x-axis the expected p-value. Click here for file
