# Supplementary material for: An inferential framework for biological network hypothesis tests
Source: BMC Bioinformatics. 2013 Mar 14;14:94. doi: 10.1186/1471-2105-14-94 (PMC3621801; doi:10.1186/1471-2105-14-94)
Supplement: Additional file 1: — R code. [file 1471-2105-14-94-S1.doc]

### Additional file 1 – R code

The initial four routines operate as kernel functions. Some of the functionality developed was not explicitly incorporated into the analyses discussed in the manuscript, e.g., the function new.beta. Adding additional features to D, e.g., directionality or handling a nxm graph representation, will require modifications to one or more of the following routines. Modulating the correlation networks using various thresholds or alternating between Null and Alternate Hypothesis forms was performed manually.

make.sample.ntwk converts weighted ordered pairs into separate adjacency and weight matrices. new.beta is a function to allow for additional weighting to various components. score.ntwk is a flexible routine to score the difference between two networks in terms of individual pieces/features. resample.target.delta is used to calculate node-level and nearby-neighbor node-level dissimilarities.

################################################################################

### Convert a Number-of-Nodesx3-based weighted network representation to a matrix form

# The 1^st^ column of ntwk.data is the weight for an edge between nodes x and y

# The 2^nd^ and 3^rd^ column are integer-based indicators of the nodes, e.g., the ordered pair

# (2,13) indicates an edge between nodes 2 and 13. no.nodes is the number of nodes.

# The individual edge and weight matrices are symmetric.

make.sample.ntwk <- function(ntwk.data,no.nodes){

# Initialize separate matrices to store edge indicators and weight indicators for each edge.

ans.incid <- ans.wgt <- matrix(rep(0,no.nodes^2),nrow=no.nodes, ncol=no.nodes)

# Iterate through the array and fill the matrix forms.

for (ii in 1:dim(ntwk.data)[1]){

x <- ntwk.data[ii,2]; y <- ntwk.data[ii,3]

ans.incid[y,x] <- ans.incid[x,y] <- 1

ans.wgt[y,x] <- ans.wgt[x,y] <- ntwk.data[ii,1]

}

# Combine/return the incidence and weight matrices as a single matrix.

answer <- cbind(ans.incid,ans.wgt)

answer

}

################################################################################

### Estimate a new Weight, via a beta distribution, using the mode and coefficient of variation

# Allows the user to make random perturbations to the weight settings for a given network.

# The function was developed for use with computer-based network simulations and was not

# used in any analyses.

# The resample.target.delta function calls this function. Removing this dependency will

# streamline that function. Since new.beta was unused in actual applications, performance

# improvements are expected to be marginal.

new.beta <- function(mu,cv){

mu_new <- numeric()

for (ii in 1:length(mu)){

b <- 3*mu[ii] - 1 - (1/(cv^2)) + mu[ii]/(cv^2)

c.1 <- (1-2*mu[ii])/(cv^2)

v.est <- (-b + sqrt(b^2-4*c.1))/2

w.est <- (cv^2)*v.est*((v.est-1)/mu[ii] + 3)

beta.simul <- rbeta(1,v.est,w.est)

mu_new[ii] <- ifelse(is.na(beta.simul)==TRUE,mu[ii],beta.simul)

}

mu_new

}

################################################################################

### Function to score the difference between the two networks, computes dissimilarity measure

# *.keep = 0 omits that feature. Simple indicator function mechanism that allows the user

# to easily determine which network features to compare.

# second.scale is a numerical weight used to scale the nearby neighborhood component comparison

# edge.keep = keep or discard edge comparisons

# wgt.keep = keep or discard weight comparisons

# nbhr.keep = keep or discard the nearby neighbors

# direc.keep = keep or discard directionality comparisons. Not used.

score.ntwk <- function(x,second.scale,edge.keep,wgt.keep,nbhr.keep,

direc.keep){

# Process first and second neighbors for edges. Weight and Direction handling is identical.

edge.first<-x[!is.na(x[,2]),2];edge.second<-x[!is.na(x[,5]),5]

edge.score <- edge.keep*(sum(edge.first) + nbhr.keep*sum(edge.second)*second.scale)

wgt.first<-x[!is.na(x[,3]),3];wgt.second<-x[!is.na(x[,6]),6]

wgt.score <- wgt.keep*(sum(wgt.first) + nbhr.keep*sum(wgt.second)*second.scale)

direc.first<-x[!is.na(x[,4]),4];direc.second<-x[!is.na(x[,7]),7]

direc.score <- direc.keep*(sum(direc.first) + nbhr.keep*sum(direc.second)*second.scale)

# Combine the various components of D.

score.answer <- edge.score + wgt.score + direc.score

score.answer

}

#############################################################################

### Score the Resample-Target difference with a coin flip at node level

# tgt, denotes network1. redraw is network2. *.incid are the 1-0 incidence matrices.

# *.wgt are the weight matrices.

# Implemented/unused features: coin - a parameter used to determine which percentage

# of weights should be perturbed with random noise, coef.var set the coefficient of variation

# of the random perturbation, and add.noise was a logical variable to define whether or not to

# perform the perturbation. These unused features were explored in a pure simulation capacity.

resample.target.delta <- function(tgt.incid,tgt.wgt,redraw.incid,

redraw.wgt,coin,coef.var,add.noise){

count.nodes <- dim(redraw.incid)[1]

delta.ntwk.resamp <- matrix(rep(0,count.nodes*7),nrow=count.nodes,ncol=7)

for (jj in 1:count.nodes){

# Add a random perturbation? Coin = 0 was always passed in to the function.

coin.flip <- ifelse(runif(1)<=coin,1,0)

coin.incid <- redraw.incid; coin.wgt <- redraw.wgt

if(coin.flip==1){coin.incid<-tgt.incid;coin.wgt<-tgt.wgt}

delta.ntwk.resamp[jj,1] <- jj

delta.ntwk.resamp[jj,2] <- sum(coin.incid[jj,]!=tgt.incid[jj,])

new.coin.wgt <- coin.wgt[jj,]

# Where the weight reassignment occurs. add.noise was always set to False.

if (add.noise==TRUE){

coin.wgt.sign <- sign(coin.wgt[jj,])

coin.wgt.mag <- abs(coin.wgt[jj,])

new.coin.wgt<-coin.wgt.sign*new.beta(coin.wgt.mag,coef.var)

}

delta.ntwk.resamp[jj,3] <- sum(abs(new.coin.wgt - tgt.wgt[jj,]))

logic.keep<-(coin.incid[jj,]==tgt.incid[jj,])&(coin.incid[jj,]==1)

second.cols <- seq(1:count.nodes)[logic.keep]

# Process the nearby neighbors

delta.second <- c(0,0,0)

for (jk in 1:length(second.cols)){

#flip coin at nearest node level; default to Redraw ntwk

coin.flip <- ifelse(runif(1)<=coin,1,0)

coin.incid <- redraw.incid; coin.wgt <- redraw.wgt

if(coin.flip==1){coin.incid<-tgt.incid;coin.wgt<-tgt.wgt}

delta.second[1]<-delta.second[1]+sum(

coin.incid[second.cols[jk],]!=tgt.incid[second.cols[jk],])

new.coin.wgt <- coin.wgt[second.cols[jk],]

if (add.noise==TRUE){

coin.wgt.sign <- sign(coin.wgt[second.cols[jk],])

coin.wgt.mag <- abs(coin.wgt[second.cols[jk],])

new.coin.wgt<-coin.wgt.sign*new.beta(coin.wgt.mag, coef.var)

}

ifelse(length(second.cols)>0,nbhr.scale<-abs(coin.wgt[jj,

second.cols[jk]]),nbhr.scale<-0)

delta.second[2]<-delta.second[2]+nbhr.scale*sum(abs(

new.coin.wgt[-jj] - tgt.wgt[second.cols[jk],-jj]))

}

delta.ntwk.resamp[jj,5:7] <- delta.second

}

delta.ntwk.resamp

}

#########################################################################

One-sample simulation code for an Erdos-Renyi random graph

library(statnet)

### Multiple Network For-loop Simulation

# Number of experiments to simulate

number.expt <- 100

# Matrix to store results

ntwk.rank.pcnt <- matrix(nrow=number.expt,ncol=4)

for (hh in 1:number.expt){

# Set the number of nodes and the probability of an edge between 2 nodes.

no.nodes <- 25

true.density <- 0.2

### Generate a TRUE network

# Set the Bernoulli parameter at 20%

true<-network(no.nodes, directed=FALSE, density=true.density)

true.ntwk<-as.matrix(true,matrix.type = "edgelist")

true.ntwk<-cbind(rep(0,dim(true.ntwk)[1]),true.ntwk[,2],true.ntwk[,1])

# Convert array-based form to matrix form; duplicate for edges and weights.

convert.to.ntwk <- make.sample.ntwk(true.ntwk,no.nodes)

tgt.incid <- convert.to.ntwk[,1:no.nodes]

tgt.wgt <- convert.to.ntwk[,-(1:no.nodes)]

# Generate an ALTERNATE network sample

# Set the Bernoulli parameter at 25%

alternate.density <- 0.25

### Generate NULL Sample incidence network

sample.B <- network(no.nodes, directed=FALSE, density=true.density)

sample.ntwk <- as.matrix(sample.B,matrix.type = "edgelist")

sample.ntwk <- cbind(rep(0,dim(sample.ntwk)[1]),sample.ntwk[,2], sample.ntwk[,1])

convert.to.ntwk <- make.sample.ntwk(sample.ntwk,no.nodes)

sample.incid <- convert.to.ntwk[,1:no.nodes]

sample.wgt <- convert.to.ntwk[,-(1:no.nodes)]

### Generate ALTERNATE Sample network based on *.DENSITY choice

sample.B.alt<-network(no.nodes,directed=FALSE,density=alternate.density)

sample.ntwk.alt <- as.matrix(sample.B.alt,matrix.type = "edgelist")

sample.ntwk.alt <- cbind(rep(0,dim(sample.ntwk.alt)[1]), sample.ntwk.alt[,2],sample.ntwk.alt[,1])

convert.to.ntwk <- make.sample.ntwk(sample.ntwk.alt,no.nodes)

sample.incid.alt <- convert.to.ntwk[,1:no.nodes]

sample.wgt.alt <- convert.to.ntwk[,-(1:no.nodes)]

### Calculate difference between Sample and Target networks

# The option below processed the edges and nearby neighbor edges. Nearby neighbors were scaled

# by exp(-2). Since coin=0 and add.noise=FALSE the coef.var = 0.4 passed into new.beta is unused.

# Used to evaluate Type I error.

stat.samp.ntwk <- score.ntwk(resample.target.delta(tgt.incid,tgt.wgt,

sample.incid,sample.wgt,0,0.4,add.noise=FALSE),

exp(-2),edge.keep=1,wgt.keep=0,nbhr.keep=1,direc.keep=0)

# Alternate hypothesis. Useful for power calculation.

stat.samp.ntwk.alt <- score.ntwk(resample.target.delta(tgt.incid,

tgt.wgt,sample.incid.alt,sample.wgt.alt,0,0.4,add.noise=FALSE),

exp(-2),edge.keep=1,wgt.keep=0,nbhr.keep=1,direc.keep=0)

# Look at Type I error and power where the nearby neighbors are excluded.

stat.samp.ntwk.nn <- score.ntwk(resample.target.delta(tgt.incid,tgt.wgt,

sample.incid,sample.wgt,0,0.4,add.noise=FALSE),

exp(-2),edge.keep=1,wgt.keep=0,nbhr.keep=0,direc.keep=0)

stat.samp.ntwk.alt.nn <- score.ntwk(resample.target.delta(tgt.incid,

tgt.wgt,sample.incid.alt,sample.wgt.alt,0,0.4,add.noise=FALSE),

exp(-2),edge.keep=1,wgt.keep=0,nbhr.keep=0,direc.keep=0)

### Resample Loop to establish a p-value for each experiment. 1000 resamples are drawn.

# Code explicitly recycles the earlier outline to generate various incidence and weight networks.

resample.no <- 1000

resample.results <- matrix(nrow=resample.no,ncol=3)

for (k in 1:resample.no){

# TRUE.DENSITY draws w/o coin flips

redraw <- network(no.nodes, directed=FALSE, density=true.density)

redraw.ntwk <- as.matrix(redraw,matrix.type = "edgelist")

redraw.ntwk <- cbind(rep(0,dim(redraw.ntwk)[1]),redraw.ntwk[,2], redraw.ntwk[,1])

redraw.ntwk <- make.sample.ntwk(redraw.ntwk,no.nodes)

redraw.incid <- redraw.ntwk[,1:no.nodes]

redraw.wgt <- redraw.ntwk[,-(1:no.nodes)]

resample.delta.ntwk <- resample.target.delta(tgt.incid,tgt.wgt,

redraw.incid,redraw.wgt,0,0.4,add.noise=FALSE)

resample.results[k,1] <- score.ntwk(resample.delta.ntwk,exp(-2),

edge.keep=1,wgt.keep=0,nbhr.keep=1,direc.keep=0)

resample.results[k,2] <- score.ntwk(resample.delta.ntwk,exp(-2),

edge.keep=1,wgt.keep=0,nbhr.keep=0,direc.keep=0)

}

# Close multiple network for loop

# Estimate the p-values under the Type I/Power and Neighbor/No Neighbor situations.

est.p.value <- (rank(c(stat.samp.ntwk,resample.results[,1]))[1])/resample.no

ntwk.rank.pcnt[hh,1] <- ifelse(est.p.value>1,1,est.p.value)

est.p.value <- (rank(c(stat.samp.ntwk.alt,resample.results[,1]))[1])/resample.no

ntwk.rank.pcnt[hh,2] <- ifelse(est.p.value>1,1,est.p.value)

est.p.value <- (rank(c(stat.samp.ntwk.nn,resample.results[,2]))[1])/resample.no

ntwk.rank.pcnt[hh,3] <- ifelse(est.p.value>1,1,est.p.value)

est.p.value <- (rank(c(stat.samp.ntwk.alt.nn,resample.results[,2]))[1])/resample.no

ntwk.rank.pcnt[hh,4] <- ifelse(est.p.value>1,1,est.p.value)

}

colnames(ntwk.rank.pcnt) <- c("TRUE.DENSITY","ALTERNATE.DENSITY","NULL.NN","ALT.NN")

# Produce the graphic illustrating the results.

par(mfrow=c(2,2))

plot(seq(1:100)/100,sort(1-ntwk.rank.pcnt[,1]),xlab="EXPECTED",

ylab="OBSERVED",main=expression(paste("(a) ",p == p[0]," and ",c[ij]==e^-2)),pch=16)

abline(0,1)

plot(seq(1:100)/100,sort(1-ntwk.rank.pcnt[,2]),xlab="EXPECTED",

ylab="OBSERVED",main=expression(paste("(b) ",p == 0.25," and ",c[ij]==e^-2)),pch=16)

abline(h=0.05)

plot(seq(1:100)/100,sort(1-ntwk.rank.pcnt[,3]),xlab="EXPECTED",

ylab="OBSERVED",main=expression(paste("(c) ",p == p[0]," and ",c[ij]==0)),pch=16)

abline(0,1)

plot(seq(1:100)/100,sort(1-ntwk.rank.pcnt[,4]),xlab="EXPECTED",

ylab="OBSERVED",main=expression(paste("(d) ",p == 0.25," and ",c[ij]==0)),pch=16)

abline(h=0.05)

#############################################################################

One-sample correlation network simulation study

library(clusterGeneration)

# result.matrix contains the p-values plotted

set.seed(12321) # Set random seed for reproducibility

cor.threshold <- 0.2 # Edge threshold; simulated correlation blocks entries > 0.2

### Create two unequal correlation networks

corr.sizes <- c(5,5,5,5,5,5) # 6 blocks of 5x5 matrices

# How many variables & nonoverlapping blocks

corr.dim <- sum(corr.sizes)

corr.lngth <- length(corr.sizes)

# Initialize resulting matrices and pointer

corr.data1 <- matrix(rep(0,corr.dim^2),nrow=corr.dim)

corr.data2 <- corr.data1

pointer.1 <- 1

# For a fixed percentage replace blocks with different corrmatrix. Used for alternate hypothesis case.

# runif() will accomplish this

# Flip back and forth between 2 and 3 at the tail end

# 10% of the correlation blocks should differ for a minimum of 1 block.

nonnull.pcnt <- 0.1; nonnull.ind <- 0

for (j in 1:corr.lngth){

corr.piece.size <- corr.sizes[j]

pointer.2 <- pointer.1+corr.piece.size-1

# Prevent isolates from appearing

make.it <- 0

while(make.it == 0){

# Generate a random correlation matrix sub-block. Test for usability or discard/retry.

temp.corr1 <- rcorrmatrix(corr.piece.size,alphad=0.1)

ifelse(min(abs(temp.corr1[lower.tri(temp.corr1)]))< cor.threshold, make.it <- 0, make.it <- 1)}

temp.corr2 <- temp.corr1

rnd.draw <- runif(1)

if(rnd.draw < nonnull.pcnt) {nonnull.ind <- 1; temp.corr2 <-

rcorrmatrix(corr.piece.size,alphad=0.1)}

# Make sure that at least one block differs between two matrices

if((j==corr.lngth)&(nonnull.ind==0)) {temp.corr2 <- rcorrmatrix(corr.piece.size,alphad=0.1)}

corr.data1[pointer.1:pointer.2,pointer.1:pointer.2]<- temp.corr1

corr.data2[pointer.1:pointer.2,pointer.1:pointer.2]<- temp.corr2

pointer.1 <- pointer.1 + corr.piece.size

}

# Comment out the line below to simulate H1 case. As written, this overwrites corr.data2 for null case.

corr.data2 <- corr.data1

### Initialize experiment and storage parameters

n.expts <- 100 # Number of individual experiments

n.data <- 200 # Sample size per group

cor.threshold <- 0.2 # Correlation block threshold

# As in Erdos-Renyi graph case, create a matrix to store the results for with and

# without neighbor comparisons.

result.matrix <- matrix(nrow=n.expts,ncol=2)

result.matrix <- as.data.frame(result.matrix)

colnames(result.matrix) <- c("Neighbor","NoNeighbor")

# Iterate through the experiments

for (hh in 1:n.expts){

# Standard multivariate normal simulation under the separate correlation structures.

normals <- mvrnorm(n.data,rep(0,dim(corr.data1)[1]),corr.data1)

diabetic <- mvrnorm(n.data,rep(0,dim(corr.data2)[1]),corr.data2)

### Generate a TRUE network

# Simulated data is used to estimate the target network; a direct threshold limit is applied to set edges.

true.pcor <- cor(normals)

cor.omit <- abs(true.pcor) < cor.threshold

true.pcor[cor.omit] <- 0

# Create a 'correlation' network for use with observation resamples

# tgt.incid.bs and tgt.wgt.bs are based on original 'correlation' network

true.bs <- true.pcor

diag(true.bs) <- 0

tgt.wgt.bs <- true.bs

cor.keep <- true.bs != 0

true.bs[cor.keep] <- 1

tgt.incid.bs <- true.bs

### Estimate DIABETIC incidence and weight networks

estimated.pcor <- cor(diabetic)

cor.omit <- abs(estimated.pcor) < cor.threshold

estimated.pcor[cor.omit] <- 0

diag(estimated.pcor) <- 0

sample.wgt <- estimated.pcor

cor.keep <- estimated.pcor != 0

estimated.pcor[cor.keep] <- 1

sample.incid <- estimated.pcor

### Calculate difference between Sample and Target networks

# As mentioned throughout, coin=0, coef.var=0.4, and add.noise=FALSE.

# Here, the edges are discarded and D is calculated with and without the nearby neighbor information.

stat.samp.ntwk.n <- score.ntwk(resample.target.delta(tgt.incid.bs,

tgt.wgt.bs,sample.incid,sample.wgt,0,0.4,add.noise=FALSE),

exp(0),edge.keep=0,wgt.keep=1,nbhr.keep=1,direc.keep=0)

stat.samp.ntwk.nn <- score.ntwk(resample.target.delta(tgt.incid.bs,

tgt.wgt.bs,sample.incid,sample.wgt,0,0.4,add.noise=FALSE),

exp(0),edge.keep=0,wgt.keep=1,nbhr.keep=0,direc.keep=0)

### Resample Loop

# Use 1000 resamples to determine the sampling distribution to compute the p-values.

# Actual network generation occurs inside the loop.

# Converting these routines to functions could easily allow for more compact code.

resample.no <- 1000

resample.results <- matrix(nrow=resample.no,ncol=2)

for (k in 1:resample.no){

# Resample from normal observations

boots.obs <- sample(seq(1:n.data),n.data,replace=TRUE)

data.sim1 <- normals[boots.obs,]

bs.estimated.cor <- cor(data.sim1)

cor.omit <- abs(bs.estimated.cor) < cor.threshold

bs.estimated.cor[cor.omit] <- 0

diag(bs.estimated.cor) <- 0

bs.sample.wgt <- bs.estimated.cor

cor.keep <- bs.estimated.cor != 0

bs.estimated.cor[cor.keep] <- 1

bs.sample.incid <- bs.estimated.cor

resample.delta.ntwk <- resample.target.delta(tgt.incid.bs,

tgt.wgt.bs,bs.sample.incid,bs.sample.wgt,0,0.4,add.noise=FALSE)

resample.results[k,1] <- score.ntwk(resample.delta.ntwk,exp(0),

edge.keep=0,wgt.keep=1,nbhr.keep=1,direc.keep=0)

resample.results[k,2] <- score.ntwk(resample.delta.ntwk,exp(0),

edge.keep=0,wgt.keep=1,nbhr.keep=0,direc.keep=0)

}

# Estimate the resample p-values.

est.p.value <- (rank(c(stat.samp.ntwk.n,resample.results[,1]))[1])/resample.no

result.matrix[hh,1] <- ifelse(est.p.value>1,0,1 - est.p.value)

est.p.value <- (rank(c(stat.samp.ntwk.nn,resample.results[,2]))[1])/resample.no

result.matrix[hh,2] <- ifelse(est.p.value>1,0,1 - est.p.value)

}

############################################################

One-sample correlation network using Normal and DM2 data

library(MASS)

library(Matrix)

# Read in raw microarray expression data once, remove column of NAs

setwd("C:/Documents and Settings/Desktop/DiabetesNtwk")

diab.data <-read.table("rawdata.txt",header=TRUE,as.is=T,sep="\t", quote="")

log.diab <- log2(diab.data[,-1])

diab.data.log <- cbind(diab.data[,1],log.diab)

# Read in subset of pathways to analyze

setwd("C:/Documents and Settings/Desktop/DiabetesNtwk/all_pathways/all_pathways")

dirlist <- dir()

geneset_length<-length(dirlist)

# Create matrix to store results. The column names suggested that pathway dimension, unique

# definition for probes per pathway, the number of probes that matched the pathway definition

# may vary. A correlation-based parametric resample was also demonstrated with a more standard

# resample approach in the original dissertation.

result.matrix <- matrix(nrow=geneset_length,ncol=6)

result.matrix <- as.data.frame(result.matrix)

colnames(result.matrix) <- c("pathwayN","uniqueN","matchN","NormalCorr","Pname","NormalBS")

# Start iteration through the list of pathways

for (hh in 1:geneset_length){

pathway <-read.delim(dirlist[hh],header=F,as.is=T,sep="\t")

result.matrix[hh,5] <- dirlist[hh]

result.matrix[hh,1] <- dim(pathway)[1]

pathway <- unique(pathway)

result.matrix[hh,2] <- dim(pathway)[1]

matchem <- match(t(pathway), diab.data.log[,1])

diab.subset <- diab.data.log[matchem[!is.na(matchem)],]

result.matrix[hh,3] <- no.nodes <- nrow(diab.subset)

diab.subset <- t(diab.subset[,-1])

# The array data was structured so that separating the Normal from Diabetics was trivial.

normals <- diab.subset[1:17,]

diabetic <- diab.subset[18:34,]

n.data <- 17 # The sample sizes were balanced here.

cor.threshold <- 0.65 # The correlation threshold for establishing a network edge.

### Generate a TRUE network

# The basic outline here is near identical to the earlier multivariate normal simulation.

true.pcor <- cor(normals)

cor.omit <- abs(true.pcor) < cor.threshold

true.pcor[cor.omit] <- 0

# Create a 'correlation' network for use with observation resamples

# tgt.incid.bs/tgt.wgt.bs are based on original 'correlation' network

true.bs <- true.pcor

diag(true.bs) <- 0

tgt.wgt.bs <- true.bs

cor.keep <- true.bs != 0

true.bs[cor.keep] <- 1

tgt.incid.bs <- true.bs

# Create 'correlation' ntwk where estimated correlation ntwk is forced

# to be positive definite. Note - based on the corr level the matrix

# may already be sparse/positive definite. This was not presented in BMC Bioinformatics.

make.pd <- nearPD(true.pcor,corr=T)

# The above true.pcor is now the PD version!

# To be safe, this needs to be converted into a correlation network.

true.pcor <- as.matrix(make.pd$mat)

true.ntwk <- true.pcor

cor.omit <- abs(true.ntwk) < cor.threshold

true.ntwk[cor.omit] <- 0

diag(true.ntwk) <- 0

tgt.wgt <- true.ntwk

cor.keep <- true.ntwk != 0

true.ntwk[cor.keep] <- 1

tgt.incid <- true.ntwk

### Estimate DIABETIC incidence and weight networks

estimated.pcor <- cor(diabetic)

cor.omit <- abs(estimated.pcor) < cor.threshold

estimated.pcor[cor.omit] <- 0

diag(estimated.pcor) <- 0

sample.wgt <- estimated.pcor

cor.keep <- estimated.pcor != 0

estimated.pcor[cor.keep] <- 1

sample.incid <- estimated.pcor

### Calculate difference between Sample and Target networks

# coin=0, coef.var = 0.4, add.noise=FALSE

# Edges are excluded, nearby neighbors are weighted by exp(0)=1. Hence the estimated weight

# is used to scale the nearby neighbors.

stat.samp.ntwk.bs <- score.ntwk(resample.target.delta(tgt.incid.bs,

tgt.wgt.bs,sample.incid,sample.wgt,0,0.4,add.noise=FALSE),

exp(0),edge.keep=0,wgt.keep=1,nbhr.keep=1,direc.keep=0)

stat.samp.ntwk <- score.ntwk(resample.target.delta(tgt.incid,tgt.wgt,

sample.incid,sample.wgt,0,0.4,add.noise=FALSE),

exp(0),edge.keep=0,wgt.keep=1,nbhr.keep=1,direc.keep=0)

### Resample Loop used to estimate resample p-values.

resample.no <- 1000

resample.results <- matrix(nrow=resample.no,ncol=2)

for (k in 1:resample.no){

# Draw from estimated (biased) nearPD correlation matrix.

# Results are only demonstrated in dissertation associated with this manuscript.

boots.obs <- mvrnorm(n.data,mu = rep(0,dim(true.pcor)[1]), Sigma = true.pcor)

re.estimated.cor <- cor(boots.obs)

cor.omit <- abs(re.estimated.cor) < cor.threshold

re.estimated.cor[cor.omit] <- 0

diag(re.estimated.cor) <- 0

re.sample.wgt <- re.estimated.cor

cor.keep <- re.estimated.cor != 0

re.estimated.cor[cor.keep] <- 1

re.sample.incid <- re.estimated.cor

resample.delta.ntwk <- resample.target.delta(tgt.incid,tgt.wgt,

re.sample.incid,re.sample.wgt,0,0.4,add.noise=FALSE)

resample.results[k,1] <- score.ntwk(resample.delta.ntwk,exp(0),

edge.keep=0,wgt.keep=1,nbhr.keep=1,direc.keep=0)

# Resample from normal observations.

# Assume Normal observations serve as basis for population.

boots.obs <- sample(seq(1:n.data),n.data,replace=TRUE)

data.sim1 <- normals[boots.obs,]

bs.estimated.cor <- cor(data.sim1)

cor.omit <- abs(bs.estimated.cor) < cor.threshold

bs.estimated.cor[cor.omit] <- 0

diag(bs.estimated.cor) <- 0

bs.sample.wgt <- bs.estimated.cor

cor.keep <- bs.estimated.cor != 0

bs.estimated.cor[cor.keep] <- 1

bs.sample.incid <- bs.estimated.cor

resample.delta.ntwk<-resample.target.delta(tgt.incid.bs,tgt.wgt.bs,

bs.sample.incid,bs.sample.wgt,0,0.4,add.noise=FALSE)

resample.results[k,2] <- score.ntwk(resample.delta.ntwk,exp(0),

edge.keep=0,wgt.keep=1,nbhr.keep=1,direc.keep=0)

}

est.p.value <- (rank(c(stat.samp.ntwk,resample.results[,1]))[1])/resample.no

result.matrix[hh,4] <- ifelse(est.p.value>1,0,1 - est.p.value)

est.p.value <- (rank(c(stat.samp.ntwk.bs,resample.results[,2]))[1])/resample.no

result.matrix[hh,6] <- ifelse(est.p.value>1,0,1 - est.p.value)

}

### Correlation Analysis: Manually pieced together results

# Code to generate the graphic from a file of stored results.

setwd("C:/Documents and Settings/Desktop/DiabetesNtwk")

corr.rslts <-read.table("DiabetesCorrResults.txt",header=TRUE,as.is=T, sep=",",quote="")

par(mfrow=c(2,2))

plot(corr.rslts[,6],corr.rslts[,14],xlab="No Edge/Neighbor",

ylab="Edge/Neighbor",pch=19, xlim=c(0,1), ylim=c(0,1),main="(a)")

abline(0,1)

plot(corr.rslts[,12],corr.rslts[,6],xlab="No Edge/No Neighbor",

ylab="No Edge/Neighbor",pch=19, xlim=c(0,1),ylim=c(0,1),main="(b)")

abline(0,1)

plot(corr.rslts[,12],corr.rslts[,14],xlab="No Edge/No Neighbor",

ylab="Edge/Neighbor",pch=19, xlim=c(0,1),ylim=c(0,1),main="(c)")

abline(0,1)

#################################################################################

Two-sample H0 and H1 partial correlation network simulation code

This code is very similar to the one-sample code. Some o f the terms, e.g., ‘normals’ and ‘diabetic’, are reused since the two-sample labels are arbitrary and portions of the code were recycled. Obvious exceptions are the resampling routine employed (the two-sample setting allows samples to be exchanged rather than simulating from a fixed distribution), the GeneNet commands used to generate the partial correlation networks, and careful control of the random number seeds. As mentioned in the manuscript, a series of random seeds were needed to generate 100 individual experiments.

GeneNet under H0

library(MASS)

library(clusterGeneration)

library(GeneNet)

# piece.together:=matrix of results cobbled together using various seeds

# The seeds are processed in order

#set.seed(64566767) # Valid 2

#set.seed(87834547) # Valid 2

#set.seed(56756745) # Valid 2

#set.seed(125765) # Valid 14

#set.seed(646294) # Valid 2

#set.seed(4128) # Valid 8

#set.seed(42984) # Valid 1

#set.seed(8582) # Valid 2

#set.seed(237843) # Valid 16

#set.seed(827434) # Valid 2

#set.seed(76832) # Valid 1

#set.seed(2) # Valid 8

#set.seed(22458) # Valid 7

#set.seed(783222) # Valid 2

#set.seed(31112) # Valid 5

#set.seed(4326790) # Valid 2

#set.seed(32792864) # Valid 4

#set.seed(876532) # Valid 2

#set.seed(422411) # Valid 3

#set.seed(67581) # Valid 9

#set.seed(12345678) # Valid 3

set.seed(555555) # Valid 3

# Need this here to control the creation of the matrices

cor.threshold <- 0.2

### Initialize number of experiments, sample sizes, number of nodes, and storage parameters

n.expts <- 25

n.data <- 200

no.nodes <- 30

result.matrix <- matrix(nrow=n.expts,ncol=2)

result.matrix <- as.data.frame(result.matrix)

colnames(result.matrix) <- c("Neighbor","NoNeighbor")

# Iterate through the experiments

for (hh in 1:n.expts){

### Create two unequal correlation networks for simulation purposes.

corr.sizes <- c(5,5,5,5,5,5)

# How many variables & nonoverlapping blocks

corr.dim <- sum(corr.sizes)

corr.lngth <- length(corr.sizes)

# Initialize resulting matrices and pointer

corr.data1 <- matrix(rep(0,corr.dim^2),nrow=corr.dim)

corr.data2 <- corr.data1

pointer.1 <- 1

nonnull.pcnt <- 0.1; nonnull.ind <- 0

for (j in 1:corr.lngth){

corr.piece.size <- corr.sizes[j]

pointer.2 <- pointer.1+corr.piece.size-1

# Prevent isolates from appearing

make.it <- 0

while(make.it == 0){

temp.corr1 <- rcorrmatrix(corr.piece.size,alphad=0.1)

ifelse(min(abs(temp.corr1[lower.tri(temp.corr1)]))< cor.threshold, make.it <- 0, make.it <- 1)}

temp.corr2 <- temp.corr1

rnd.draw <- runif(1)

if(rnd.draw < nonnull.pcnt) {nonnull.ind <- 1; temp.corr2 <-

rcorrmatrix(corr.piece.size,alphad=0.1)}

# Make sure that at least one block differs between the two matrices

if((j==corr.lngth)&(nonnull.ind==0)) {temp.corr2 <- rcorrmatrix(corr.piece.size,alphad=0.1)}

corr.data1[pointer.1:pointer.2,pointer.1:pointer.2]<- temp.corr1

corr.data2[pointer.1:pointer.2,pointer.1:pointer.2]<- temp.corr2

pointer.1 <- pointer.1 + corr.piece.size

}

# Comment out the line below for H1 (alternate hypothesis) case

corr.data2 <- corr.data1

# Create the 2 samples using the Normal/Diabetic recycled labels.

normals <- mvrnorm((1*n.data),rep(0,dim(corr.data1)[1]),corr.data1)

diabetic <- mvrnorm(n.data,rep(0,dim(corr.data2)[1]),corr.data2)

# Combine the data into one large dataset

data.sim <- rbind(normals,diabetic)

### Generate a TRUE network via GeneNet

# Correlation-to-partial correlation conversion

true.pcor <- cor2pcor(cor(normals))

true.test.results <- ggm.test.edges(true.pcor,plot=FALSE) # Test edges

true.ntwk <- extract.network(true.test.results, cutoff.ggm=0.5) # Extract network

true.ntwk <- true.ntwk[,1:3]

convert.to.ntwk <- make.sample.ntwk(true.ntwk,no.nodes) # Convert form for use here.

tgt.incid <- convert.to.ntwk[,1:no.nodes]

tgt.wgt <- convert.to.ntwk[,-(1:no.nodes)]

### Estimate DIABETIC incidence and weight networks

estimated.pcor <- cor2pcor(cor(diabetic))

sample.test.results <- ggm.test.edges(estimated.pcor,plot=FALSE)

sample.ntwk <- extract.network(sample.test.results, cutoff.ggm=0.5)

sample.ntwk <- sample.ntwk[,1:3]

convert.to.ntwk <- make.sample.ntwk(sample.ntwk,no.nodes)

sample.incid <- convert.to.ntwk[,1:no.nodes]

sample.wgt <- convert.to.ntwk[,-(1:no.nodes)]

### Calculate difference between Sample and Target networks

# coin=0, coef.var=0.4, add.noise=FALSE

# Edges are excluded, nearby neighbors are excluded or weighted by exp(0)=1.

# When included, the estimated weight is used to scale the nearby neighbors.

stat.samp.ntwk.n <- score.ntwk(resample.target.delta(tgt.incid,tgt.wgt,

sample.incid,sample.wgt,0,0.4,add.noise=FALSE),

exp(0),edge.keep=0,wgt.keep=1,nbhr.keep=1,direc.keep=0)

stat.samp.ntwk.nn <- score.ntwk(resample.target.delta(tgt.incid,tgt.wgt,

sample.incid,sample.wgt,0,0.4,add.noise=FALSE),

exp(0),edge.keep=0,wgt.keep=1,nbhr.keep=0,direc.keep=0)

### Resample Loop to estimate p-values

resample.no <- 1000

resample.results <- matrix(nrow=resample.no,ncol=2)

for (k in 1:resample.no){

# Resample from normal observations

# Random reshuffles of the Sample labels more easily facilitates computing the

# sampling distribution for the two-sample case.

boots.series <- seq(1:(2*n.data))

boots.obs1 <- sample(boots.series,n.data,replace=FALSE)

data.sim1 <- data.sim[boots.obs1,]

data.sim2 <- data.sim[-boots.obs1,]

bs.estimated.pcor1 <- cor2pcor(cor(data.sim1))

bs.estimated.pcor2 <- cor2pcor(cor(data.sim2))

bs.sample.test.results1 <- ggm.test.edges(bs.estimated.pcor1,plot=FALSE)

bs.sample.test.results2 <- ggm.test.edges(bs.estimated.pcor2, plot=FALSE)

bs.sample.ntwk1 <- extract.network(bs.sample.test.results1, cutoff.ggm=0.5)

bs.sample.ntwk2 <- extract.network(bs.sample.test.results2, cutoff.ggm=0.5)

bs.sample.ntwk1 <- bs.sample.ntwk1[,1:3]

bs.sample.ntwk2 <- bs.sample.ntwk2[,1:3]

bs.convert.to.ntwk1 <- make.sample.ntwk(bs.sample.ntwk1,no.nodes)

bs.convert.to.ntwk2 <- make.sample.ntwk(bs.sample.ntwk2,no.nodes)

bs.sample.incid1 <- bs.convert.to.ntwk1[,1:no.nodes]

bs.sample.wgt1 <- bs.convert.to.ntwk1[,-(1:no.nodes)]

bs.sample.incid2 <- bs.convert.to.ntwk2[,1:no.nodes]

bs.sample.wgt2 <- bs.convert.to.ntwk2[,-(1:no.nodes)]

resample.delta.ntwk <- resample.target.delta(bs.sample.incid1,

bs.sample.wgt1,bs.sample.incid2,bs.sample.wgt2,0,0.4,add.noise=FALSE)

resample.results[k,1] <- score.ntwk(resample.delta.ntwk,exp(0),

edge.keep=0,wgt.keep=1,nbhr.keep=1,direc.keep=0)

resample.results[k,2] <- score.ntwk(resample.delta.ntwk,exp(0),

edge.keep=0,wgt.keep=1,nbhr.keep=0,direc.keep=0)

}

# Close multiple network for loop

est.p.value <- (rank(c(stat.samp.ntwk.n,resample.results[,1]))[1])/resample.no

result.matrix[hh,1] <- ifelse(est.p.value>1,0,1 - est.p.value)

est.p.value <- (rank(c(stat.samp.ntwk.nn,resample.results[,2]))[1])/resample.no

result.matrix[hh,2] <- ifelse(est.p.value>1,0,1 - est.p.value)

}

piece.together <- rbind(piece.together,result.matrix[1:3,])

# Produce graphic.

par(lwd=2)

plot(seq(1:100)/100,sort(piece.together[,1]),xlab="BULLET - NEIGHBOR,

CROSS - NO NEIGHBOR",ylab="P-VALUE", pch=16,xlim=c(0,1),ylim=c(0,1))

par(new=TRUE)

plot(seq(1:100)/100,sort(piece.together[,2]),ann=FALSE,axes=FALSE,pch=3)

abline(0,1)

GeneNet under H1

Only the random number seeds/plotting section are supplied. Removing a single line, documented in the previous section, produces data under the alternate hypothesis.

#set.seed(2) # Valid 8

#set.seed(125765) # Valid 2

#set.seed(8582) # Valid 1

#set.seed(22458) # Valid 6

#set.seed(42984) # Valid 10

#set.seed(76832) # Valid 2

#set.seed(31112) # Valid 5

#set.seed(783222) # Valid 6

#set.seed(646294) # Valid 15

#set.seed(422411) # Valid 3

#set.seed(1029) # Valid 9

#set.seed(67581) # Valid 3

#set.seed(4326790) # Valid 4

#set.seed(827434) # Valid 2

#set.seed(32792864) # Valid 4

#set.seed(876532) # Valid 4

#set.seed(237843) # Valid 1

#set.seed(12345678) # Valid 3

#set.seed(4128) # Valid 12

par(lwd=2)

plot(piece.together100[,1],piece.together100[,2],xlab="NEIGHBOR",

ylab="NO NEIGHBOR", pch=19,xlim=c(0,1),ylim=c(0,1))

abline(0,1)

##################################################################################

Analysis of the ovarian cancer dataset

This routine builds extensively on the previous routines. Simple edits were performed to compute the numerous phenotypic comparisons.

library(MASS)

library(GeneNet)

### Initialize experiment and storage parameters

result.matrix <- matrix(nrow=10,ncol=2)

result.matrix <- as.data.frame(result.matrix)

colnames(result.matrix) <- c("Neighbor","NoNeighbor")

setwd("H:/GEO_Data")

allraw <- read.table("DataSubsetforR.csv" , header = TRUE, sep = ",", row.names = 1)

# Rows 1-5: Cell cycle - G1/S

# Rows 6-18: Cell cycle - S/G2

# Rows 19-24: Checkpoints

# Rows 25-29: DNA damage repair

# Rows 30-42: DNA synthesis and replication

# Gene names will be converted to row names

# Cols SBT - 1:11, SCA1 - 12:21, SCA3 - 22:36

# Add the row centering and transpose the raw data

geneavg <- apply(allraw,1,mean)

allraw <- sweep(allraw,1,geneavg); allraw <- t(allraw)

# Subset the 3 phenotypes compared.

SBT <- allraw[1:11,]; SCA1 <- allraw[12:21,]; SCA3 <- allraw[22:36,]

# Break the transposed data into gene sets

SBT_G1S<-SBT[,1:5];SCA1_G1S<-SCA1[,1:5];SCA3_G1S<-SCA3[,1:5]

SBT_SG2<-SBT[,6:18];SCA1_SG2<-SCA1[,6:18];SCA3_SG2<-SCA3[,6:18]

SBT_Check<-SBT[,19:24];SCA1_Check<-SCA1[,19:24];SCA3_Check<-SCA3[,19:24]

SBT_Repair<-SBT[,25:29];SCA1_Repair<-SCA1[,25:29]

SCA3_Repair<-SCA3[,25:29]

SBT_SynRepl<-SBT[,30:42];SCA1_SynRepl<-SCA1[,30:42]

SCA3_SynRepl<-SCA3[,30:42]

# There are 15 pairings to potentially investigate: SBT to SCA1, SBT to SCA3, SCA1 to SCA3

# Comparisons below are used for nontrivial tests once the estimated networks are known.

# Using GGM cutoff = 0.5.

# G1S

SBT_use <- SBT_G1S; SCA1_use <- SCA1_G1S; SCA3_use <- SCA3_G1S

phenotype1 <- SCA1_use; phenotype2 <- SCA3_use; total.n <- 25; phen1.n <- 10; hh <- 1; no.nodes <- 5

# SG2 1

#SBT_use <- SBT_SG2; SCA1_use <- SCA1_SG2; SCA3_use <- SCA3_SG2

#phenotype1 <- SBT_use; phenotype2 <- SCA1_use; total.n <- 21; phen1.n <- 11; hh <- 2; no.nodes <- 13

# SG2 2

#SBT_use <- SBT_SG2; SCA1_use <- SCA1_SG2; SCA3_use <- SCA3_SG2

#phenotype1 <- SCA1_use; phenotype2 <- SCA3_use; total.n <- 25; phen1.n <- 10; hh <- 3; no.nodes <- 13

# Check 1

#SBT_use <- SBT_Check; SCA1_use <- SCA1_Check; SCA3_use <- SCA3_Check

#phenotype1 <- SBT_use; phenotype2 <- SCA1_use; total.n <- 21; phen1.n <- 11; hh <- 4; no.nodes <- 6

# Check 2

#SBT_use <- SBT_Check; SCA1_use <- SCA1_Check; SCA3_use <- SCA3_Check

#phenotype1 <- SCA1_use; phenotype2 <- SCA3_use; total.n <- 25; phen1.n <- 10; hh <- 5; no.nodes <- 6

# Repair

#SBT_use <- SBT_Repair; SCA1_use <- SCA1_Repair; SCA3_use <- SCA3_Repair

#phenotype1 <- SBT_use; phenotype2 <- SCA1_use; total.n <- 21; phen1.n <- 11; hh <- 6; no.nodes <- 5

# SynRep1

#SBT_use<-SBT_SynRepl;SCA1_use<-SCA1_SynRepl;SCA3_use<-SCA3_SynRepl

#phenotype1 <- SBT_use; phenotype2 <- SCA1_use; total.n <- 21; phen1.n <- 11; hh <- 7; no.nodes <- 13

# SynRep2

#SBT_use<-SBT_SynRepl;SCA1_use<-SCA1_SynRepl;SCA3_use<-SCA3_SynRepl

#phenotype1 <- SCA1_use; phenotype2 <- SCA3_use; total.n <- 25; phen1.n <- 10; hh <- 8; no.nodes <- 13

# Combine the data into one large dataset

data.sim <- rbind(phenotype1,phenotype2)

### Estimate First Phenotype network

true.pcor <- cor2pcor(cor(phenotype1))

true.test.results <- network.test.edges(true.pcor,plot=FALSE)

true.ntwk <- extract.network(true.test.results, cutoff.ggm=0.5)

true.ntwk <- true.ntwk[,1:3]

convert.to.ntwk <- make.sample.ntwk(true.ntwk,no.nodes)

tgt.incid <- convert.to.ntwk[,1:no.nodes]

tgt.wgt <- convert.to.ntwk[,-(1:no.nodes)]

### Estimate Second Phenotype network

estimate1.pcor <- cor2pcor(cor(phenotype2))

sample.test.results <- network.test.edges(estimate1.pcor,plot=FALSE)

sample.ntwk <- extract.network(sample.test.results, cutoff.ggm=0.5)

sample.ntwk <- sample.ntwk[,1:3]

convert.to.ntwk <- make.sample.ntwk(sample.ntwk,no.nodes)

sample.incid <- convert.to.ntwk[,1:no.nodes]

sample.wgt <- convert.to.ntwk[,-(1:no.nodes)]

### Calculate difference between Sample and Target networks

# coin=0, coef.var=0.4, add.noise=FALSE

# Edges are excluded, nearby neighbors are excluded or weighted by exp(0)=1.

# When included, the estimated weight is used to scale the nearby neighbors. Only the with-neighbor

# data is presented here.

stat.samp.ntwk.n <- score.ntwk(resample.target.delta(tgt.incid,tgt.wgt,

sample.incid,sample.wgt,0,0.4,add.noise=FALSE),

exp(0),edge.keep=0,wgt.keep=1,nbhr.keep=1,direc.keep=0)

stat.samp.ntwk.nn <- score.ntwk(resample.target.delta(tgt.incid,tgt.wgt,

sample.incid,sample.wgt,0,0.4,add.noise=FALSE),

exp(0),edge.keep=0,wgt.keep=1,nbhr.keep=0,direc.keep=0)

### Resample Loop

resample.no <- 1000

resample.results <- matrix(nrow=resample.no,ncol=2)

for (k in 1:resample.no){

# Resample from normal observations

boots.series <- seq(1:total.n)

boots.obs1 <- sample(boots.series,phen1.n,replace=FALSE)

data.sim1 <- data.sim[boots.obs1,]

data.sim2 <- data.sim[-boots.obs1,]

bs.estimated.pcor1 <- cor2pcor(cor(data.sim1))

bs.estimated.pcor2 <- cor2pcor(cor(data.sim2))

bs.sample.test.results1 <- ggm.test.edges(bs.estimated.pcor1,plot=FALSE)

bs.sample.test.results2 <- ggm.test.edges(bs.estimated.pcor2,plot=FALSE)

bs.sample.ntwk1 <- extract.network(bs.sample.test.results1, cutoff.ggm=0.5)

bs.sample.ntwk2 <- extract.network(bs.sample.test.results2, cutoff.ggm=0.5)

bs.sample.ntwk1 <- bs.sample.ntwk1[,1:3]

bs.sample.ntwk2 <- bs.sample.ntwk2[,1:3]

bs.convert.to.ntwk1 <- make.sample.ntwk(bs.sample.ntwk1,no.nodes)

bs.convert.to.ntwk2 <- make.sample.ntwk(bs.sample.ntwk2,no.nodes)

bs.sample.incid1 <- bs.convert.to.ntwk1[,1:no.nodes]

bs.sample.wgt1 <- bs.convert.to.ntwk1[,-(1:no.nodes)]

bs.sample.incid2 <- bs.convert.to.ntwk2[,1:no.nodes]

bs.sample.wgt2 <- bs.convert.to.ntwk2[,-(1:no.nodes)]

resample.delta.ntwk <- resample.target.delta(bs.sample.incid1,

bs.sample.wgt1,bs.sample.incid2,bs.sample.wgt2,0,0.4, add.noise=FALSE)

resample.results[k,1] <- score.ntwk(resample.delta.ntwk,exp(0),

edge.keep=0,wgt.keep=1,nbhr.keep=1,direc.keep=0)

resample.results[k,2] <- score.ntwk(resample.delta.ntwk,exp(0),

edge.keep=0,wgt.keep=1,nbhr.keep=0,direc.keep=0)

}

est.p.value <- (rank(c(stat.samp.ntwk.n,resample.results[,1]))[1])/resample.no

result.matrix[hh,1] <- ifelse(est.p.value>1,0,1 - est.p.value)

est.p.value <- (rank(c(stat.samp.ntwk.nn,resample.results[,2]))[1])/resample.no

result.matrix[hh,2] <- ifelse(est.p.value>1,0,1 - est.p.value)
