# Supplementary material for: An inferential framework for biological network hypothesis tests
Source: BMC Bioinformatics. 2013 Mar 14;14:94. doi: 10.1186/1471-2105-14-94 (PMC3621801; doi:10.1186/1471-2105-14-94)
Supplement: Additional file 2: — Ovarian cancer genes analyzed. Subset of analyzed genes as categorized by Bracken et al. [47]. [file 1471-2105-14-94-S2.doc]

### Additional file 2 – Ovarian cancer genes analyzed

Subset of analyzed genes as categorized by Bracken *et al*. [47].

| G1-S phase of the cell cycle | *MYBL2, E2F1, E2F3, CDK2, CDC25A* |
| --- | --- |
| S-G2 phase of the cell cycle | *SMC4, CKS1B, PLK1, CDC20, CDC2*, *CCNA2, NDC80, CKS2, AURKB, MKI67*, *CCNA2-2, PRC1, KIF4A* |
| Checkpoint | *MAD2L1, BUB1B, TTK, CENPE, BUB1*, *BRCA2* |
| DNA damage and repair | *RAD54L, FEN1, RAD51, BARD1, MSH2* |
| DNA synthesis and replication | *PCNA, TOP2A, MCM3, MCM6, MCM2*, *TK1, CDC6, RFC4, CDC45L, RFC3*, *POLA2, CDC7, RRM2* |
