# Supplementary figures and images for: An inferential framework for biological network hypothesis tests
Source: BMC Bioinformatics. 2013 Mar 14;14:94. doi: 10.1186/1471-2105-14-94 (PMC3621801; doi:10.1186/1471-2105-14-94)

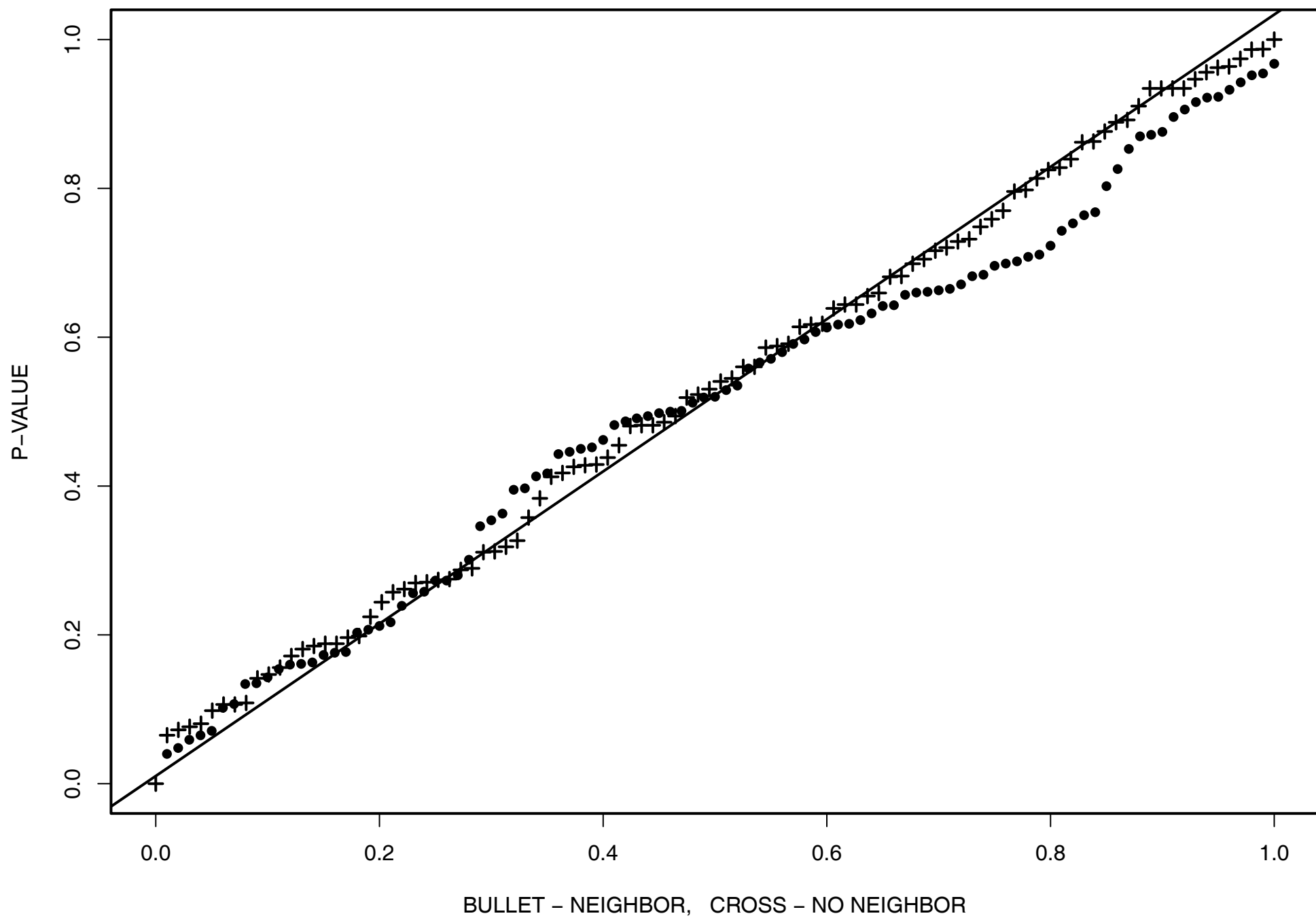

Supplement: Additional file 3: — Two-sample comparison for partial correlation networks under H0. A uniform qq-plot of the 100 resample p-values for a test of H0 Π1= Π2 versus H1 Π1≠ Π2 under the null hypothesis. The y-axis is the observed p-value; the x-axis the expected p-value. [file 1471-2105-14-94-S3.pdf]
